# A Multimorbidity Analysis of Hospitalized Patients With COVID-19 in Northwest Italy: Longitudinal Study Using Evolutionary Machine Learning and Health Administrative Data

**DOI:** 10.2196/52353

**Published:** 2024-07-18

**Authors:** Dayana Benny, Mario Giacobini, Alberto Catalano, Giuseppe Costa, Roberto Gnavi, Fulvio Ricceri

**Affiliations:** 1 Centre for Biostatistics, Epidemiology, and Public Health Department of Clinical and Biological Sciences University of Turin Turin Italy; 2 Modeling and Data Science Department of Mathematics University of Turin Turin Italy; 3 Data Analysis and Modeling Unit Department of Veterinary Sciences University of Turin Turin Italy; 4 Department of Translational Medicine University of Piemonte Orientale Novara Italy; 5 Unit of Epidemiology Regional Health Service Local Health Unit Torino 3 Turin Italy

**Keywords:** machine learning, evolutionary algorithm, multimorbidity, data analysis, epidemiology, feature bins, COVID-19, long COVID, ICD, ATC, polypharmacy, sparse binary data, feature engineering, public health, severity, epidemiology, coronavirus, SARS-CoV-2, risk assessments, risk assessment, data, data mining, big data, longitudinal study, longitudinal analysis, longitudinal analyses, health data, Italy

## Abstract

**Background:**

Multimorbidity is a significant public health concern, characterized by the coexistence and interaction of multiple preexisting medical conditions. This complex condition has been associated with an increased risk of COVID-19. Individuals with multimorbidity who contract COVID-19 often face a significant reduction in life expectancy. The postpandemic period has also highlighted an increase in frailty, emphasizing the importance of integrating existing multimorbidity details into epidemiological risk assessments. Managing clinical data that include medical histories presents significant challenges, particularly due to the sparsity of data arising from the rarity of multimorbidity conditions. Also, the complex enumeration of combinatorial multimorbidity features introduces challenges associated with combinatorial explosions.

**Objective:**

This study aims to assess the severity of COVID-19 in individuals with multiple medical conditions, considering their demographic characteristics such as age and sex. We propose an evolutionary machine learning model designed to handle sparsity, analyzing preexisting multimorbidity profiles of patients hospitalized with COVID-19 based on their medical history. Our objective is to identify the optimal set of multimorbidity feature combinations strongly associated with COVID-19 severity. We also apply the Apriori algorithm to these evolutionarily derived predictive feature combinations to identify those with high support.

**Methods:**

We used data from 3 administrative sources in Piedmont, Italy, involving 12,793 individuals aged 45-74 years who tested positive for COVID-19 between February and May 2020. From their 5-year pre–COVID-19 medical histories, we extracted multimorbidity features, including drug prescriptions, disease diagnoses, sex, and age. Focusing on COVID-19 hospitalization, we segmented the data into 4 cohorts based on age and sex. Addressing data imbalance through random resampling, we compared various machine learning algorithms to identify the optimal classification model for our evolutionary approach. Using 5-fold cross-validation, we evaluated each model’s performance. Our evolutionary algorithm, utilizing a deep learning classifier, generated prediction-based fitness scores to pinpoint multimorbidity combinations associated with COVID-19 hospitalization risk. Eventually, the Apriori algorithm was applied to identify frequent combinations with high support.

**Results:**

We identified multimorbidity predictors associated with COVID-19 hospitalization, indicating more severe COVID-19 outcomes. Frequently occurring morbidity features in the final evolved combinations were age>53, R03BA (glucocorticoid inhalants), and N03AX (other antiepileptics) in cohort 1; A10BA (biguanide or metformin) and N02BE (anilides) in cohort 2; N02AX (other opioids) and M04AA (preparations inhibiting uric acid production) in cohort 3; and G04CA (Alpha-adrenoreceptor antagonists) in cohort 4.

**Conclusions:**

When combined with other multimorbidity features, even less prevalent medical conditions show associations with the outcome. This study provides insights beyond COVID-19, demonstrating how repurposed administrative data can be adapted and contribute to enhanced risk assessment for vulnerable populations.

## Introduction

### Background

COVID-19, classified as a highly infectious disease, poses a severe threat to vulnerable populations, making it a critical public health concern and a significant global epidemiological issue. The first Italian case of COVID-19 was diagnosed in the Lombardy region on February 21, 2020. The virus quickly spread across the country, leading to a nationwide lockdown and overwhelming the health care system. Italy was among the countries hardest hit by the COVID-19 pandemic, with Piedmont, a region in the northwest, experiencing a high number of cases during the first wave.

Multimorbidity refers to the presence of multiple coexisting medical conditions in a patient, which interact with each other, resulting in a complex and multidimensional health condition [1]. At the population level, it has been established that interactions between diseases can increase the severity of the overall medical condition and complicate the treatment of other diseases within the combination [2,3]. In people infected with SARS-CoV-2, multimorbidity can increase the severity of the infection [4,5]. Therefore, it is important to identify specific disease combinations that could impact the severity of COVID-19 in individuals with multimorbidity.

It is necessary to point out that having one or more of these chronic health conditions does not guarantee severe COVID-19 development, but it does increase the risk. Different diseases may affect COVID-19 outcomes differently. Therefore, identifying specific disease combinations and studying interactions between different chronic health conditions are essential for understanding the severity of COVID-19 among individuals with multimorbidity. This can help health care professionals identify those at the highest risk of severe complications and provide appropriate prevention, care, and treatment.

Studying multimorbidity using traditional methods can be labor-intensive, requiring the identification of high-dimensional combinatorial features. Also, there is no universally accepted list of medical conditions to define multimorbidity. To address these challenges, efforts must focus on identifying low-dimensional representations of multimorbidity features for effective outcome prediction. High-order input features make machine learning models more prone to overfitting, and identifying meaningful high-order combinatorial features requires extensive effort from experts with domain knowledge.

Contrary to misconceptions, the concept of multimorbidity analysis in patients with COVID-19 is not outdated. Our study introduces a cutting-edge tool designed to analyze the complex interactions among diverse chronic health conditions and their collective impact, which could be valuable in situations similar to recent health crises.

Traditionally, research on multimorbidity has focused on counting the total number of chronic conditions rather than considering individual experiences and the effects of different combinations of diseases [6]. Count-based measures of multimorbidity have been utilized to predict emergency hospitalizations [7,8]. Common combinations of medical conditions have been documented to delineate patterns of multimorbidity [9,10]. Previous studies have explored multimorbidity combinations using methods such as latent class analysis [11], cluster analysis [12], network analysis [13], factor analysis [14], association rules, and tree-based analysis [13,15].

Rare features, such as diseases and drugs with low occurrence rates in the data, can pose significant challenges for both statistical and machine learning analyses. Their lower prevalence in the data can result in sparsity, which may lead to poorer predictions.

Some studies using machine learning to investigate multimorbidity patterns tackled data set sparsity by strategies such as removing sparsity-inducing features [16], consolidating feature categories after one-hot encoding [17], or clustering rare features [18]. However, while these methods can alleviate sparsity, they may also result in the loss of important information and impede the meaningful interpretation of multimorbidity features [19].

### Importance of the Proposed Method for Multimorbidity Research

With the increasing prevalence of electronic health records and other large data sets, there is a rising demand for efficient and effective methods to analyze and comprehend multimorbidity. By utilizing machine learning algorithms and other advanced computational techniques, researchers can gain deeper insights into the underlying mechanisms and risk factors associated with multimorbidity. This understanding can significantly inform more effective prevention and treatment strategies.

An evolutionary algorithm coupled with deep learning–based feature scoring represents a powerful approach for analyzing multimorbidity data [20]. This method involves multiple steps aimed at identifying the most relevant features for predicting the target variable while minimizing the number of features used.

Initially, the data set undergoes preprocessing by using a feature binning approach to generate various subsets or bins of the multimorbidity features. This step aims to reduce sparsity in the data and facilitates more effective feature scoring. Subsequently, deep learning is utilized to score the features within each subset based on their relevance for predicting the target variable. The result of this process is a feature score assigned to each feature within every subset. Next, an evolutionary algorithm is applied to select the optimal subset of features based on their scores. The algorithm initiates by generating a population of candidate feature subsets and iteratively enhances this population through selection, crossover, and mutation operations [21]. The fitness of each candidate solution is assessed using a fitness function that integrates the deep learning–based feature scores derived from each subset or bin of features.

The output of the evolutionary algorithm is a subset of features that are most relevant for predicting the target variable. These features can be utilized for further analysis, such as constructing a predictive model or uncovering the underlying associations of multimorbidity patterns. In summary, using an evolutionary algorithm with deep learning–based feature scoring offers a robust approach to analyzing multimorbidity data, pinpointing the most influential features for predicting the target variable. This approach can result in enhanced model performance, faster training times, and improved interpretability in complex data sets featuring multimorbidity [22].

### Study Goal

This study aims to identify multimorbidity patterns that may serve as predictors of COVID-19 hospitalization (as a proxy for a more severe COVID-19 outcome) using evolutionary algorithms. To assess the effectiveness of our approach, we conducted a comparative analysis to justify the use of deep learning as a classifier over other established machine learning algorithms in terms of prediction accuracy. The evolutionary model may excel not only because of its superior predictive performance but also because it effectively manages sparsity. This capability could result in better identification of key features, more stable predictions, or enhanced performance within specific subgroups of the data [23].

A logistic model might reveal multimorbidity patterns that exhibit complexity[24]. However, linear models, despite their high interpretability, may struggle in sparse data sets where effective feature selection is relevant [25]. In such cases, evolutionary algorithms, particularly genetic algorithms, excel by adeptly handling feature interactions with complexity and identifying optimal feature subsets, a task that proves challenging for linear models in sparse data scenarios [26].

The selected models are interpreted using Shapley Additive Explanations (SHAP) values [27] to understand the relationship between the multimorbidity features across different cohort data and hospitalization outcomes. The proposed method also generates a feature-engineered data set containing a specified number of outcome-associated combinations or bins of multimorbidity. Then, the best performing bins are analyzed to explore the frequency of various multimorbidity patterns across all cohorts.

This study demonstrates how our innovative tool has the potential to revolutionize traditional risk assessment approaches. By incorporating complex combinations of diseases, the tool aims to improve the accuracy of predicting severe outcomes for individuals with multiple chronic conditions. With its adaptable design, it ensures applicability even in evolving scenarios involving different communicable diseases, highlighting its ongoing relevance. This study focuses on investigating the complexities of disease interactions, demonstrating how our tool could reshape risk assessment in similar contexts.

## Methods

### Study Design

This retrospective cohort study is designed to exhaustively examine the presence or absence of various multimorbidities in patients over a 5-year period leading up to the onset of COVID-19. The core of our analysis is the longitudinal tracking of these multimorbidities, relevant for understanding their impact on subsequent health outcomes, particularly hospitalizations due to COVID-19. Central to the study’s design is its longitudinal nature, involving systematic analysis of patient data collected over a specified time frame to assess how individual and collective health conditions influence the risk and severity of COVID-19–related hospitalizations. The retrospective cohort framework enables the use of existing medical records, including hospital discharge summaries and drug prescription data, to construct a comprehensive picture of each patient’s health status in the years leading up to the pandemic. Through an analysis of these long-term health patterns, our aim is to understand how preexisting conditions influence the severity of COVID-19.

This study involves examining individuals’ multimorbidity history over a 5-year period, encompassing both pre– and post–COVID-19 diagnosis periods. It further investigates the relationship between multimorbidity history, COVID-19 positivity, and the subsequent severity of COVID-19. This design allows for observing participants over an extended time frame and evaluating outcomes not only before but also after the critical event of COVID-19 diagnosis.

### Multimorbidity Data Set

Data for the multimorbidity analyses were collected from the Piedmont Longitudinal Study (PLS), a health administrative cohort comprising anonymized records linked at the individual level from various social, health, and administrative databases [5,19]. Since February 2020, the PLS has been augmented by the regional COVID-19 platform, which collects data on COVID-19 infections. From these databases, we utilized registers for (1) hospital discharges, (2) drug prescription data, and (3) COVID-19 hospitalizations of individuals diagnosed with SARS-CoV-2 infection for the first time between February 22, 2020, and May 31, 2020. We retrieved the 5-year medical history of patients positive for COVID-19 from these data sets. The extracted data comprises 12,793 individuals aged 45-74 years who tested positive for the first time for SARS-CoV-2 infection. Our study specifically focused on this age group to eliminate potential influences from both younger and older individuals on the results. Also, this approach helped to mitigate bias associated with patients residing in nursing homes.

As the study was integrated into the National Statistical Plan, no ethical approvals or permits were required, and the database used for the analyses contained only anonymized data. Further information regarding ethical considerations and data availability can be found in the “Ethical Considerations” section.

### Ethical Considerations

This study is part of the PLS, a specific project within the Italian National Statistical Program, proposed by the National Statistical System (SISTAN), integrated into the National Statistical Program (Programma Statistico Nazionale [PSN]), an initiative endorsed by the National Institute of Statistics (Istituto Nazionale di Statistica [ISTAT]) in Italy. This project is annually approved by the Italian Parliament. Since 2003, a dedicated form (PIE-00001 “Monitoring of socio-economic differences in mortality and morbidity through longitudinal studies”) has been included in the PSN, currently effective for the 3-year period from 2020 to 2022 [28] and recently renewed for 2023-2025.

Ethical approval or permits from the ethics committee are not necessary for this research. Access to PLS data within the responsible institution does not require informed consent, as stipulated by the Presidential Decree published in the Official Gazette of the Italian Republic N. 122 of May 26, 2022, under the PSN [29].

Consequently, informed consent from an ethical committee is not required for this study. All analyses adhered to the principles of the World Medical Association’s Declaration of Helsinki, and to preserve privacy, the data used for analysis underwent deidentification.

### Construction of the Exposure’s Variables

**Figure 1:**
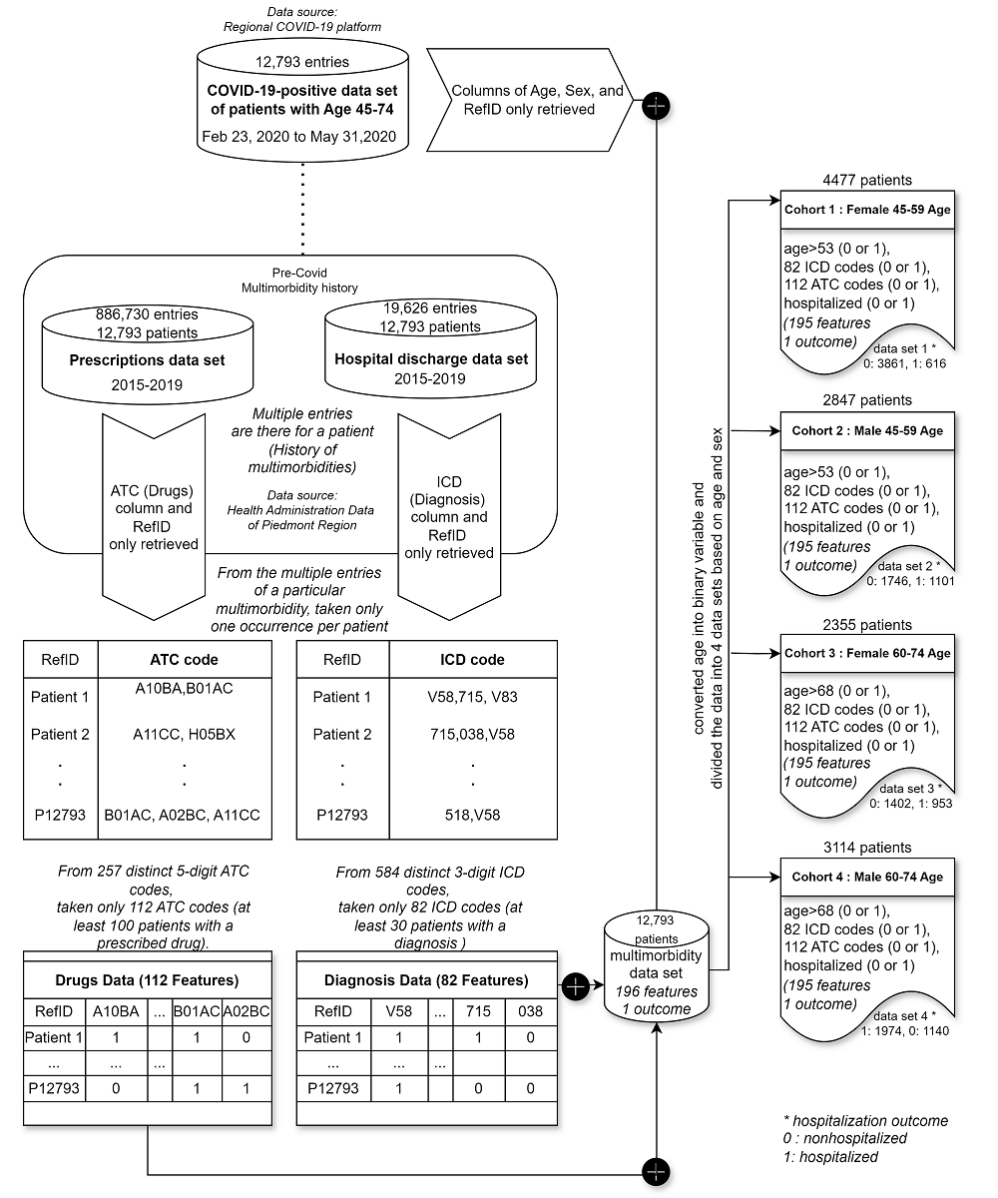
Transformation of data sets: The Multimorbidity data, derived from prescription and hospital discharge data sets, are merged with the COVID-19 database containing patients who tested positive. The resulting data set is then subdivided into 4 cohorts based on age and sex. ATC: Anatomical Therapeutic Chemical; ICD: 9th International Classification of Diseases-Clinical Modification.

In this longitudinal cohort study, patients’ multimorbidity status over the past 5 years (2015-2019) was compared in relation to a specific outcome (hospitalization due to COVID-19). Multimorbidity was defined using records from hospital discharge and drug prescription registers. In the data sets for hospital discharges and drug prescriptions, multiple entries exist for each patient with COVID-19. The drug prescriptions data set comprises approximately 1 million records, while the hospital discharges data set includes around 19,000 entries. From the drug prescriptions data set, the Anatomical Therapeutic Chemical (ATC) classification system codes were used. All distinct ATC codes up to the 4th level (the first 5 digits of the ATC codes) were considered in this study. One-hot encoding was applied to convert categorical codes into separate feature columns with binary values (0 or 1) indicating the absence or presence of drugs in each patient’s prescription history. Similarly, from the hospital discharge data, the 9th International Classification of Diseases-Clinical Modification (ICD-9-CM) codes [30] (as diagnosis codes) were used, and one-hot encoding was applied. Following these transformations, only drug codes and diagnosis codes meeting the criterion “at least 100 patients with this code in the COVID-19-positive patients’ data” are retained. Consequently, 194 features were derived from drug codes (n=112) and diagnosis codes (n=82) as multimorbidity features from the entire data set, where the presence and absence of these features are denoted as 1 and 0, respectively. Also, 2 features—age and sex—are included, with sex coded as 1 for females and 0 for males. Subsequently, the preprocessed data were segmented into 4 data sets based on age and sex. The data set transformation steps are illustrated in Figure 1.

Subsets of various cohorts were obtained by considering the study population falling within the age criteria of “aged 45-59 years” and “aged 60-74 years.” This subdivision is made because individuals aged 60 and above are often categorized as part of the older population [5]. Median values within each age range are used as threshold values for discretizing the age feature in this study. This approach involves categorizing or binning based on median values within each specified age range. For example, to discretize the age feature into groups such as “45-59” and “60-74,” we used medians (53 for “45-59” and 68 for “60-74”) as thresholds.

In the data sets for the younger cohorts (cohorts 1 and 2), the age feature was converted into a binary variable, where 1 represents age>53 and 0 represents age≤53. The age values were derived from the 2020 COVID-19 data, and the age of 53 years was used as a threshold to divide the younger population into 2 subgroups (45-53 and 54-59 years). Similarly, the older population was divided into 2 subgroups (60-68 and 69-74 years), where the age feature was converted into a binary variable, with 1 indicating age>68 and 0 indicating age≤68. All 4 cohort data sets were treated as distinct binary classification problems. The input variables, comprising multimorbidity history and age, along with the outcome variable indicating whether a patient was hospitalized due to COVID-19, were represented as binary values.

In our study, multimorbidity features included the presence and absence of prescribed drugs and diagnosed diseases, as well as patient age and sex. However, due to the rarity of many medical conditions in the study population, the resulting data set became sparse when encoding absence as 0 values.

### Data Imbalance Rectification

A significant challenge when working with clinical data is predicting rare events, which can result in an imbalance problem when the target variable has more observations in one class than in others. Therefore, it is beneficial to handle imbalanced raw data properly to prevent bias toward a particular class. All data sets used in this study exhibit imbalance, and resampling is recommended as a solution. To address this, randomly balanced samples were drawn from the unbalanced original data set to achieve class balance. Subsequently, a statistical hypothesis test, specifically the one-proportion *z*-test, was performed. This test compares the proportion of the sampled population with that of the raw data population, ensuring the representativeness of the randomly balanced sample data compared with the original cohort data set and mitigating potential biases.

The steps performed to obtain an unbiased balanced data set with significant features are as follows:

- Extract all minority and majority samples attributed to the outcome value from the original cohort data set.
- Randomly select samples belonging to the majority class so that they are equal in number to the minority class to achieve a balanced data set.
- Calculate the prevalence of each feature in the randomly selected samples and compare it with the prevalence in the original population.
- Perform a one-proportion *z*-test on all nonzero variables to assess whether the frequency distribution of a feature in the resampled data is representative of the same feature in the original cohort data set, using a significance level of .05.
- Evaluate the results of the one-proportion *z*-test, considering the test statistic and *P* values, to determine the significance and eliminate nonsignificant features from the sampled data.

### Model Development

#### Machine Learning Algorithms

**Figure 2:**
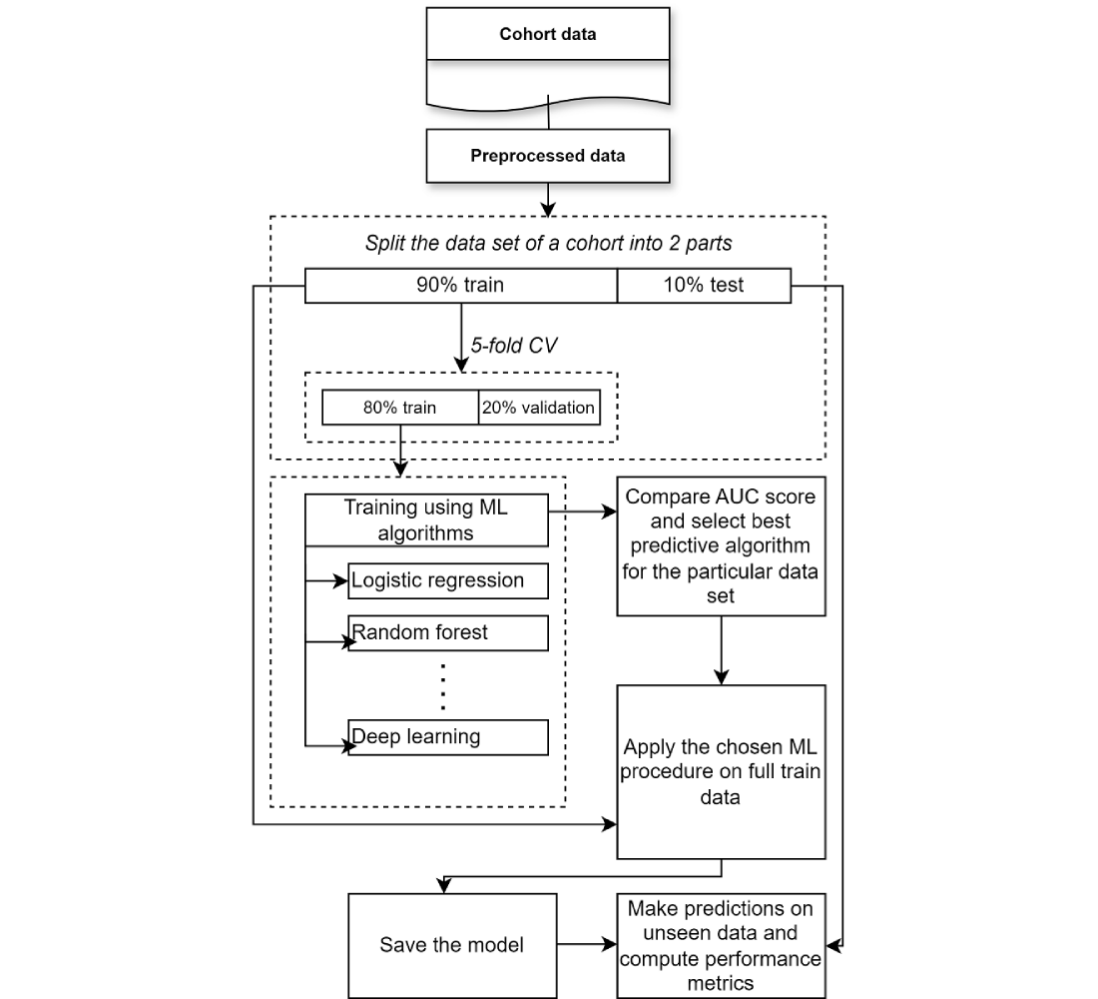
Selecting best ML model for each cohort data set: A streamlined process of selecting the optimal ML model for cohort data sets, using supervised algorithms for binary classification of multimorbidity profiles, with comparison based on a scoring grid featuring average cross-validated scores. AUC: area under the curve; ML: machine learning.

To select the best model, we evaluated the performance of various supervised machine learning algorithms. Using labeled health records enables the application of supervised learning, specifically binary classification to classify a patient’s multimorbidity profile. Deep learning and other machine learning algorithms were applied to all cohort data sets, as depicted in Figure 2. Results were compared using a scoring grid with average cross-validated scores.

#### SHAP Analysis

**Figure 3:**
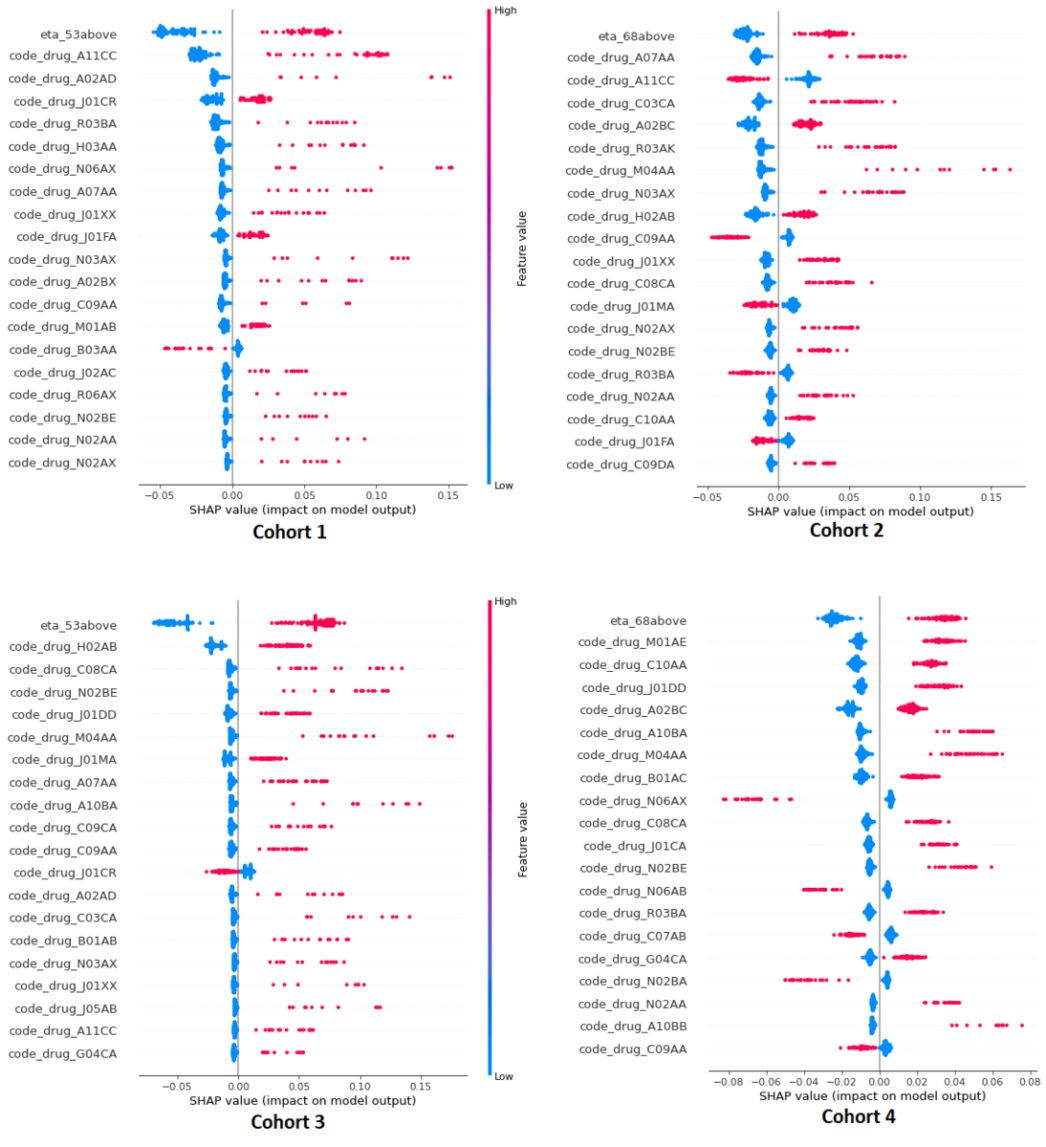
SHAP beeswarm plots illustrating the impact of all features on COVID-19 hospitalization for all 4 models. SHAP: Shapley Additive Explanations.

SHAP values were used to elucidate the contribution of individual features in predicting hospitalization outcomes across all cohorts. These SHAP values for all features were plotted, with their positions on the y-axis indicating their impact on the model outcome. Beeswarm plots of SHAP values were used to explore the distribution of influence that each feature has on the model outcome, with features of greater importance positioned higher on the graph. Each data point for a feature corresponds to a single patient, with the position of the data point (SHAP value) on the x-axis indicating the effect of that feature on the model outcome for that specific patient. In the SHAP beeswarm plots, when multimorbidity is present (indicated by a feature value of 1 in red), a higher positive SHAP value suggests that this feature acts as a risk factor for hospitalization. Conversely, a more negative SHAP value in the presence of multimorbidity indicates that this feature acts as a protective factor against hospitalization risk for the patient. These findings are illustrated in Figure 3.

#### Deep Learning With Sparse Data

This study addresses a sparse health care data set that includes rare medical conditions and drugs, posing challenges for statistical and machine learning analyses due to their low prevalence [23]. To tackle this issue, the study utilizes sequential deep learning with the Adaptive Gradient Algorithm (AdaGrad), an optimization algorithm well-suited for handling sparse data [31]. AdaGrad’s adaptive scaling of the learning rate eliminates the need for manual tuning and enhances robustness compared with stochastic gradient descent. Also, the study uses early stopping functionality to improve the model’s performance.

In all deep learning models, dropout has been used as a regularization technique to mitigate overfitting during training [32]. Specifically, a dropout layer with a 20% dropout rate has been introduced after the first and second layers in the sequential model. Given the binary classification nature of the problem, the default loss function used is binary cross-entropy loss [33].

#### Feature Selection for Discovering the Optimal Set of Multimorbidity Features

Feature selection as a preprocessing method eliminates irrelevant and redundant information, aiding in dimensionality reduction [34]. There are 3 main methods of feature selection: filter-based, embedded, and wrapper-based methods. Filter-based methods typically generate models with reduced predictive performance compared with the other 2 methods. The embedded method performs an optimal feature subset search while constructing the model, whereas the wrapper method selects the best feature subset based on the classifier’s performance. In our study, we used a wrapper method that utilizes deep learning as the classifier algorithm and an evolutionary algorithm as the search strategy to generate feature subsets (bins). The best performing bin is determined using the area under the curve (AUC) metric and selected as the optimal subset of multimorbidity features highly associated with COVID-19 hospitalization.

#### Evolutionary Machine Learning

The use of evolutionary algorithms represents a promising approach for extracting a reduced set of meaningful and accurate rare associations, particularly beneficial for addressing challenges such as sparse data, epistatic associations with features, and high-dimensional representations of features. Evolutionary machine learning is a hybrid method that leverages evolutionary computation to overcome challenges encountered in various machine learning tasks [35]. Compared with traditional algorithms that rely on exhaustive search-based techniques, evolutionary algorithms offer a more robust solution.

Several key considerations arise when performing feature engineering with evolutionary algorithms: (1) a feature’s lack of prevalence does not necessarily imply irrelevance; it could still strongly influence the outcome; (2) addressing data sparsity poses a challenge for many machine learning methods, particularly concerning features with near-0 variance; and (3) evaluating combinations of features may yield greater predictive power than assessing isolated features alone, emphasizing the significance of exploring feature interactions.

We used a genetic algorithm to create an optimized feature matrix. Initially, features were randomly grouped into bins, each forming a feature matrix. These bins were then regrouped using a genetic algorithm and a wrapper-based method interacting with a classifier. The study adopted the elitism principle to preserve the best-performing bins as checkpoints. The final feature matrix represents the evolved engineering matrix after all iterations, designed to address issues of data sparsity and incorporate interactions among various multimorbidity features.

**Figure 4:**
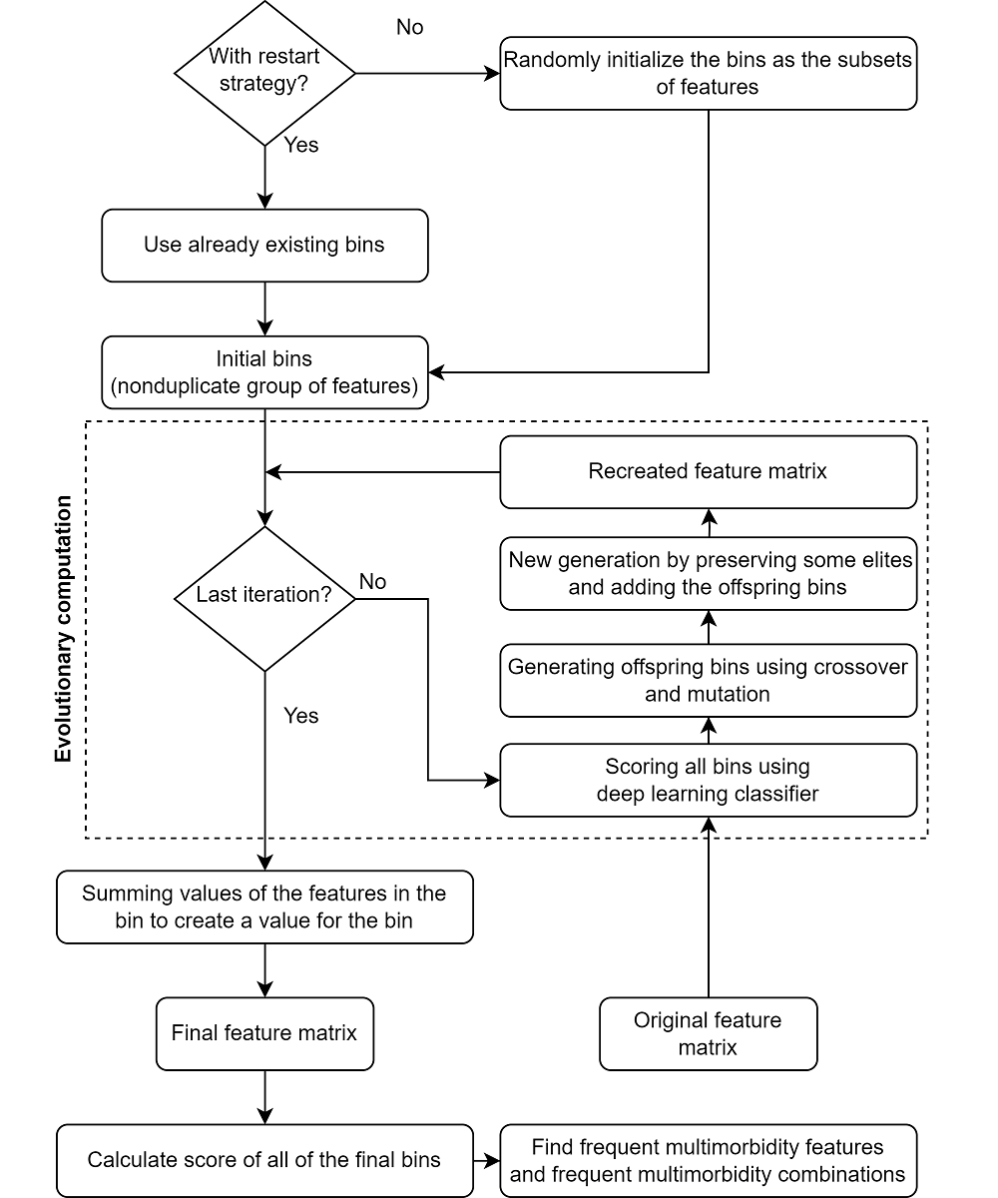
Illustration of the evolutionary approach carried out in this study: The process begins by randomly initializing subsets of features. Through the execution of evolutionary computation, a final feature matrix is generated. Subsequently, frequently occurring combinations and features are identified.

The proposed evolutionary approach in the study is an evolutionary algorithm–based wrapper method, illustrated in Figure 4. It is a modified version of an existing evolutionary algorithm known as the Relevant Association Rare-variant-bin Evolver [23]. The proposed method differs from the existing approach in several ways: it utilizes a prediction-based method with separate training and testing phases, incorporates a deep learning technique with an AdaGrad optimizer, and estimates the frequency of occurrence of specific features within the best performing feature combinations. Also, the scores produced by the deep learning model serve as fitness scores to assess the performance of multimorbidity combinations in each cycle.

#### Frequent Multimorbidity Features

The most prevalent multimorbidity combinations were identified to discern patterns among patients with COVID-19 using the Apriori algorithm. Applied to the final bins data set, which includes various multimorbidity feature combinations obtained from the evolutionary algorithm, the Apriori algorithm utilized the support measure to gauge the commonality of feature combinations across rows in the final bins. To focus on relevant feature combinations, only the most common multimorbidity patterns were analyzed. Frequent combinations of features were examined using a minimum support threshold (smin) set at 0.5 to derive frequent itemsets.

## Results

### Characteristics of the COVID-19 Population

Table 1 summarizes the characteristics of the COVID-19 population, while Table 2 presents the distribution of hospitalized and nonhospitalized patients.

**Table 1:** Characteristics of the COVID-19 population.

Demographics			Values
**Age groups (years), n/N (%)**			
	45-53^a^	4179/7324 (57.06)	
	54-59^a^	3145/7324 (42.94)	
	60-68^b^	3296/5469 (60.27)	
	69-74^b^	2173/5469 (39.73)	
**Female, n/N (%)**			
	Younger age group	4477/7324 (61.13)	
	Older age group	2355/5479 (42.98)	
**Male, n/N (%)**			
	Younger age group	2847/7324 (38.87)	
	Older age group	3114/5479 (56.84)	
**Age (years), mean (SD)**			
	Younger age group	52.3 (4.18)	
	Older age group	67 (4.55)	

^a^Considered the younger age group.

^b^Considered the older age group.

**Table 2:** Distribution of hospitalized and nonhospitalized patients with COVID-19.

Demographics			Hospitalized					Nonhospitalized					
			Male, n/N		Female, n/N		Male, n/N			Female, n/N			
**Age group (years)**													
	45-59	1101/1717		616/1717		1746/5607			3861/5607				
	45-53	522/825		303/825		1031/3354			2323/3354				
	54-59	579/892		313/892		715/2253			1538/2253				
	60-74	1974/2927		953/2927		1140/2542			1402/2542				
	60-68^a^	1073/1585		512/1585		740/1711			971/1711				
	69-74^a^	901/1342		441/1342		400/831			431/831				

^a^Considered the older age group.

### One-Proportion *z*-Test Results

The one-proportion *z*-test was conducted on all features, and the results comparing randomly sampled data with the original cohort data sets are presented in Multimedia Appendix 1.

### Performance of Machine Learning Models and Model Selection

Table 3 illustrates the performance evaluation of the deep learning model used across all 4 cohorts. The evaluation of other machine learning models is presented in Multimedia Appendix 2.

**Figure 5:**
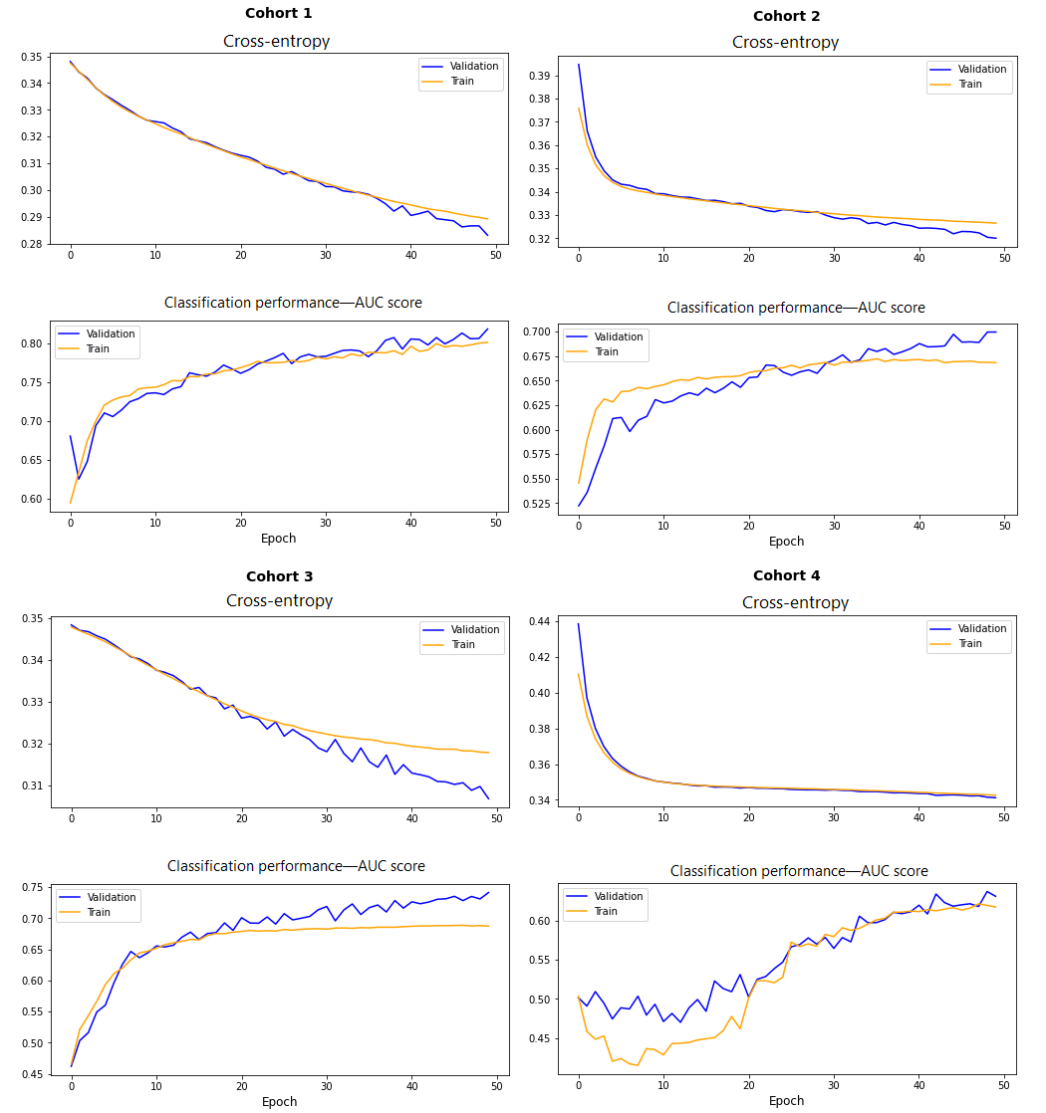
Model loss plot and AUC score over epochs—validation of model efficiency for each cohort through 2 line plots: The topmost plot depicts the binary cross-entropy loss for the epochs for the training and validation data sets, and the bottommost one presents the classification performance (AUC score) over epochs. AUC: area under the curve.

For each cohort, as depicted in Figure 5, 2 line plots were generated to validate the model’s effectiveness using cross-validation.

**Table 3:** Performance evaluation of the deep learning model.

Cohort	AUC^a^ score 5-fold CV^b^ (SD), %	Training AUC score (loss)	Test AUC score (loss)	Accuracy, %	Precision, %	Recall, %	*F*_1_-score, %
1	77 (1.87)	82% (0.28)	80% (0.29)	76	85	63	72
2	68 ( 1.94)	71% (0.30)	67% (0.32)	62	62	61	62
3	67 (1.87)	74% (0.31)	69% (0.32)	67	70	60	65
4	61 (2.44)	65% (0.34)	62% (0.34)	63	62	68	65

^a^AUC: area under the curve.

^b^CV: coefficient of variation.

In cohort 1, it is evident that the model learns the problem efficiently and rapidly, achieving an AUC score of 82% on the training data set and 80% on the test data set. The close similarity between these scores suggests that the model is neither overfitting nor underfitting. The cross-entropy loss plot showed that the model has converged, with acceptable loss values observed on both data sets. The classification performance plot further indicated convergence. The model’s performance and convergence suggested that cross-entropy loss is suitable for effectively learning this neural network problem. In cohort 2, the model achieved performance scores of 71% on the training data set and 67% on the test data set, with reasonable loss values. The minimal difference between these scores indicated that the model learned the problem satisfactorily. In cohort 3, the model achieved a training score of 74% and a test AUC score of 69%. Observing that there was no significant improvement after 30 epochs, early stopping could be implemented during model training to prevent overfitting and stabilize the validation loss. In cohort 4, although the loss plot appeared well-converged, the model showed slightly lower classification performance compared with the models in other cohorts.

### Influence of Individual Features on COVID-19 Hospitalization: Most Prevalent Multimorbidity Features in Evolved Bins

**Figure 6:**
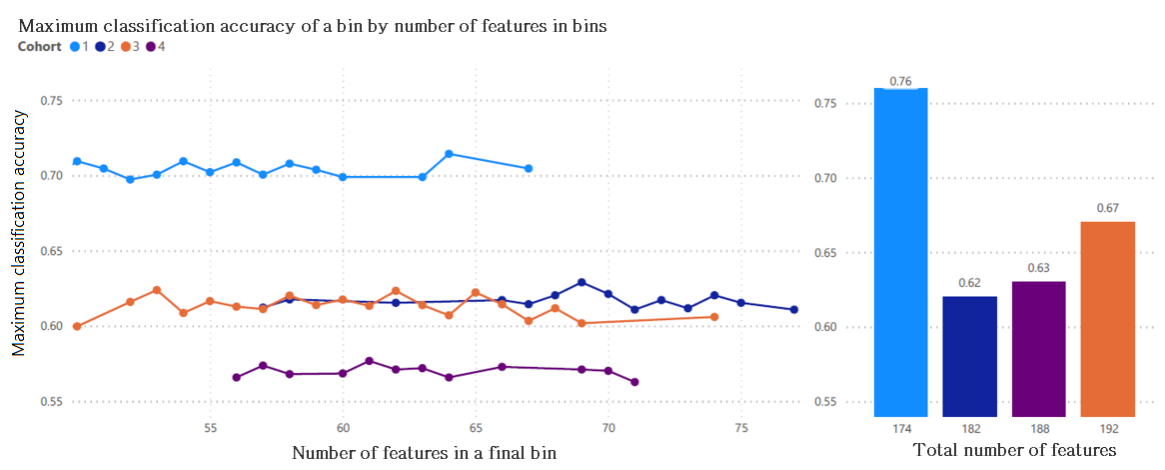
Maximum classification accuracy achieved by a bin versus number of features in that bin using evolutionary approach (left side) and the accuracy score achieved exclusively by the deep learning model (right side) with all the available features in the cohort.

The accuracy scores of the evolutionarily obtained final bins have been calculated. The highest accuracy was achieved for cohort 1 using the evolutionary approach to find outcome-associated best subsets of features, reaching 71.43% (95% CI 67.31-67.97) with 64 features. For cohort 2, the accuracy was 63% (95% CI 59.43-59.75) using 69 features. Cohort 3 achieved an accuracy of 62.38% (95% CI 59.84-60.09) with 53 features, while cohort 4 achieved an accuracy of 58% (95% CI 55.42-55.63) using 61 features. These results were then compared with the accuracy score of the deep learning model that utilized all features, as illustrated in Figure 6.

In cohort 1, frequently occurring multimorbidity features included age>53, R03BA (glucocorticoid inhalants), and N03AX (other antiepileptics). For cohort 2, A10BA (biguanide or metformin) and N02BE (anilides) were prevalent. Cohort 3 exhibited frequent occurrences of N02AX (other opioids) and M04AA (preparations inhibiting uric acid production), while G04CA (Alpha-adrenoreceptor antagonists) was notable in cohort 4.

Table 4 displays the multimorbidity features that occurred most frequently in the final bins data set across all cohorts, using a minimum support (smin) measure of 0.6. It includes the prevalence of these features in the sampled data set. Detailed statistics for all other features can be found in Multimedia Appendix 3.

**Table 4:** Frequently occurred morbidity features in the evolutionarily obtained final bins data set with support measure with corresponding P values, and the prevalence of the features in the sampled data set utilized for the predictive analysis.

Cohort, category, and features				Description			P value		Support		Prevalence
**1**											
	**Age**										
		>53	—^a^			<.001		0.84		41.15	
	**ATC#^b^**										
		R03BA			Glucocorticoids	<.001		0.85		15.5	
		N03AX			Other antiepileptics	<.001		0.82		5.6	
		R06AX			Other antihistamines for systemic use	<.001		0.79		6.74	
		J01XX			Other antibacterials	<.001		0.78		14.2	
		C03CA			Sulfonamides, plain	<.001		0.76		5.19	
		N02AX			Other opioids	<.001		0.74		6.9	
		A11CC			Vitamin D and analogs	<.001		0.73		23.05	
		C09CA			Angiotensin II receptor blockers, plain	<.001		0.69		5.44	
		J01CA			Penicillins with extended spectrum	<.001		0.66		14.12	
		J01EE			Combinations of sulfonamides and trimethoprim, including derivatives	.03		0.61		2.44	
	**ICD#^c^**										
		298			Other nonorganic psychoses	.16		0.68		0.16	
		411			Other acute and subacute forms of ischemic heart disease	.32		0.62		0.08	
**2**											
	**ATC#**										
		A10BA			Biguanides	<.001		0.86		4.31	
		N02BE			Anilides	<.001		0.79		6.4	
		J05AB			Nucleosides and nucleotides (excluding reverse transcriptase inhibitors)	<.001		0.76		2.91	
		C03CA			Sulfonamides, plain	<.001		0.76		4.09	
		M04AA			Preparations inhibiting uric acid production	<.001		0.74		5.13	
		C09CA			Angiotensin II receptor blockers, plain	<.001		0.71		8.4	
		C02CA			Alpha-adrenoreceptor antagonists	<.001		0.65		3.22	
		C08CA			Dihydropyridine derivatives	<.001		0.65		7.4	
		J02AC			Triazole and tetrazole derivatives	.03		0.64		6.18	
		N06AB			Selective serotonin reuptake inhibitors	.03		0.63		8.58	
		S01EE			Prostaglandin analogs	.07		0.62		0.68	
		N03AG			Fatty acid derivatives	.08		0.61		2.5	
		M01AB			Acetic acid derivatives and related substances	.001		0.6		18.21	
		N03AE			Benzodiazepine derivatives	.17		0.6		1.54	
	**ICD#**										
		V64			Surgical or other procedures not carried out because of contraindications	>.99		0.64		0.18	
		V54			Other orthopedic aftercare	.26		0.64		0.32	
		188			Malignant neoplasm of the bladder	.32		0.63		0.18	
		735			Acquired deformities of the toe	>.99		0.6		0.18	
		454			Varicose veins of lower extremities	.83		0.6		1.04	
		820			Fractures of the neck of the femur	.32		0.6		0.05	
**3**											
	**ATC#**										
		N02AX			Other opioids	<.001		0.84		12.96	
		M04AA			Preparations inhibiting uric acid production	<.001		0.82		8.5	
		C03EA			Low-ceiling diuretics and potassium-sparing agents	<.001		0.76		5.35	
		A02BA			H2-receptor antagonists	.004		0.75		4.04	
		B01AB			Heparin group	<.001		0.73		12.59	
		N03AX			Other antiepileptics	<.001		0.7		11.7	
		N02AA			Natural opium alkaloids	<.001		0.68		13.9	
		J05AB			Nucleosides and nucleotides (excluding reverse transcriptase inhibitors)	.008		0.65		5.77	
		A12AA			Calcium	.11		0.62		4.67	
		C07BB			Beta blocking agents, selective, and thiazides	.16		0.62		2.62	
		B03BB			Folic acid and derivatives	<.001		0.61		9.23	
		R03AC			Selective beta-2-adrenoreceptor agonists	.005		0.6		7.19	
	**ICD#**										
		295			Schizophrenic disorders	.03		0.68		0.73	
		813			Fractures of the radius and ulna	.62		0.68		0.84	
**4**											
	**ATC#**										
		G04CA			Alpha-adrenoreceptor antagonists	.02		0.8		25.75	
		J01CA			Penicillins with extended spectrum	.008		0.73		14.47	
		C09DA			Angiotensin II receptor blockers and diuretics	.07		0.66		13.11	
		C09AA			ACE inhibitors, plain	.03		0.66		26.32	
		B01AA			Vitamin K antagonists	.001		0.64		4.61	
		C03CA			Sulfonamides, plain	.002		0.62		16.49	
	**ICD#**										
		995			Certain adverse effects not elsewhere classified	>.99		0.61		0.44	

^a^Not available.

^b^ATC: Anatomical Therapeutic Chemical.

^c^ICD: 9th International Classification of Diseases.

**Figure 7:**
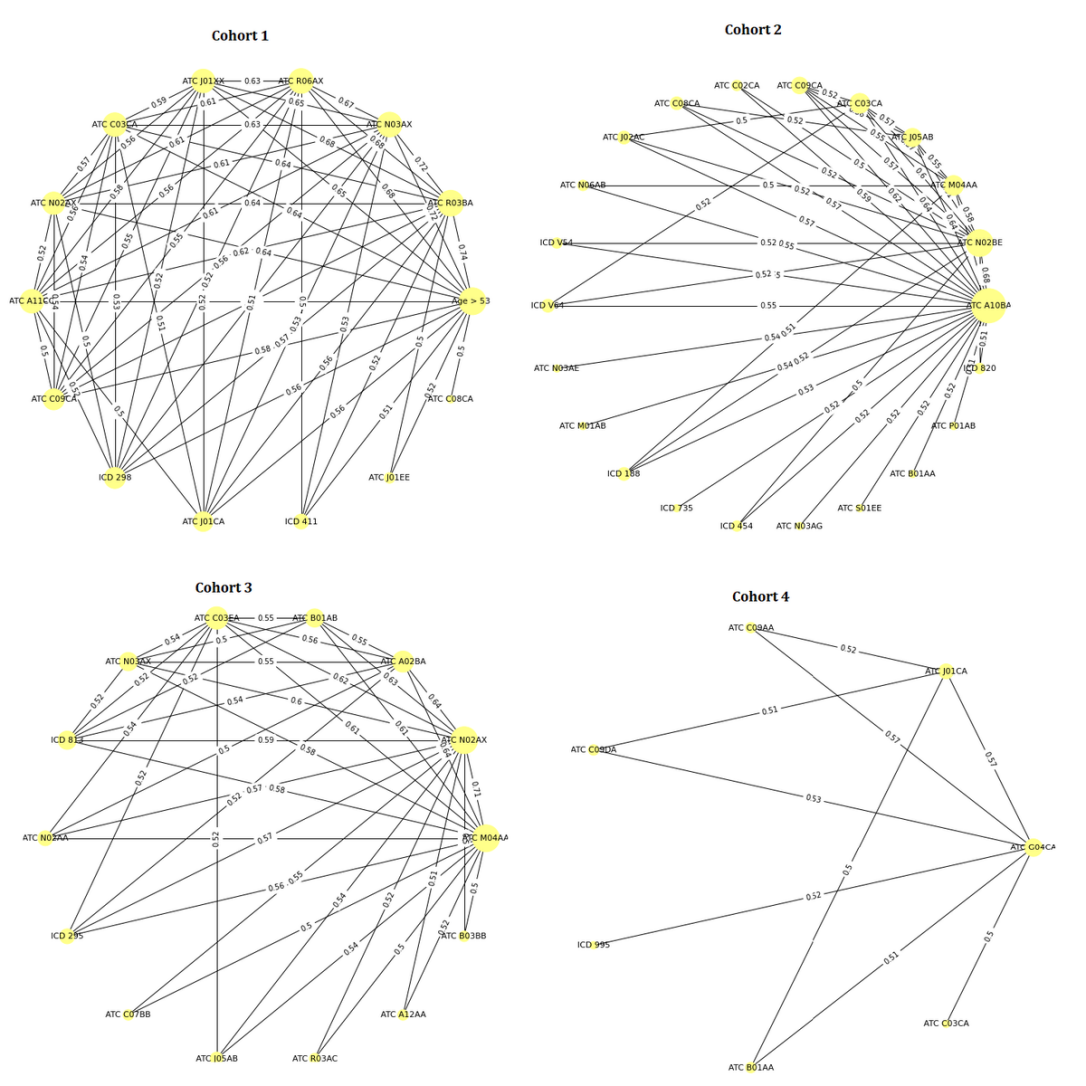
Frequent outcome-associated multimorbidity feature combinations (2 variable combinations with smin=0.5) in each cohort.

The graph in Figure 7 illustrates the combinations derived from analyzing all 2-variable combinations with a minimum support (smin) of 0.5. Detailed results for these combinations can be found in Multimedia Appendix 4.

**Figure 8:**
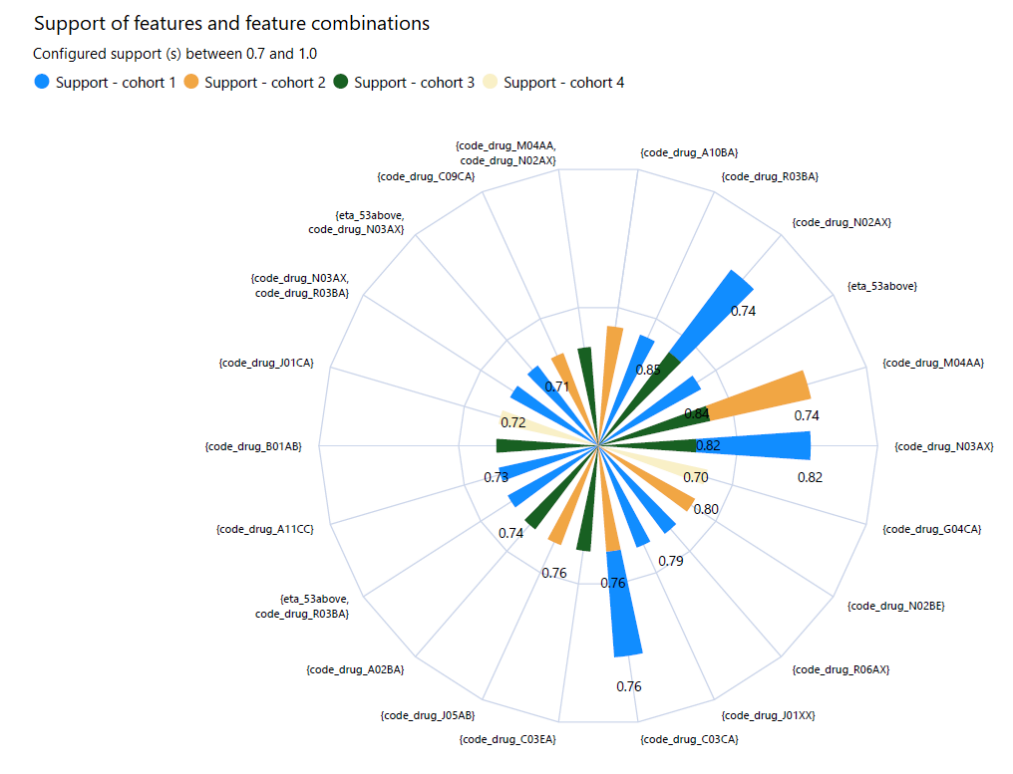
Illustration of the features and combinations that frequently appeared in the final bins data set when configuring the support (s) between 0.7 and 1.0 as radar chart, with features presented in more than 1 cohort stacked.

We observed that certain multimorbidity features appear consistently across most outcome-associated bins. Additionally, some features are common and frequent across the final bins of various cohorts. Table 5 tabulates the features and combinations that frequently appeared in the final bins data set, using a support (s) threshold between 0.7 and 1.0. These findings are graphically presented in Figure 8.

**Table 5:** Frequently appeared features and combinations in the final bins data set when the support (s) is configured between 0.7 and 1.0.

Support	Length of the combination	Frequent features	Cohort
0.85	1	ATC R03BA	1
0.84	1	Age>53	1
0.82	1	ATC N03AX	1
0.79	1	ATC R06AX	1
0.78	1	ATC J01XX	1
0.76	1	ATC C03CA	1
0.74	1	ATC N02AX	1
0.74	2	Age>53, ATC R03BA	1
0.73	1	ATC A11CC	1
0.72	2	ATC N03AX, ATC R03BA	1
0.72	2	Age>53, ATC N03AX	1
0.86	1	ATC A10BA	2
0.79	1	ATC N02BE	2
0.76	1	ATC C03CA	2
0.76	1	ATC J05AB	2
0.74	1	ATC M04AA	2
0.71	1	ATC C09CA	2
0.84	1	ATC N02AX	3
0.82	1	ATC M04AA	3
0.76	1	ATC C03EA	3
0.75	1	ATC A02BA	3
0.73	1	ATC B01AB	3
0.71	2	ATC M04AA, ATC N02AX	3
0.7	1	ATC N03AX	3
0.8	1	ATC G04CA	4
0.73	1	ATC J01CA	4

## Discussion

### Principal Findings

The primary findings of the study highlight prevalent multimorbidity patterns identified within the evolved data set. These patterns, characterized by specific ATC codes and ICD codes, show significant associations with hospitalization outcomes, particularly among distinct demographic groups. This analysis not only provides insights into COVID-19 but also suggests potential broader applications. Repurposing data originally collected for administrative purposes, this innovative approach shows promise for multimorbidity analysis in public health. It shows the adaptability and versatility of the methodology, capable of extracting valuable insights from existing data sets to inform effective public health strategies and interventions.

While our evolutionary machine learning model shows only marginal superiority compared with other prediction models, even slight improvements in predictive performance can hold significant value in real-world applications, particularly in critical fields such as health care where accuracy is mandatory. Moreover, we acknowledge that achieving the highest prediction performance may not be the sole objective of our study.

In the baseline method, variables are transformed into a binary format using one-hot encoding, which leads to the creation of a large, sparse matrix [26]. Evolutionary models typically excel in handling high-dimensional data compared with linear models, utilizing their enhanced ability to navigate and effectively utilize the search space [36]. Evolutionary approaches have drawbacks such as challenges in interpretability, computational efficiency, and a higher risk of overfitting [37]. However, despite the simplicity and clear interpretability of linear models, evolutionary models excel in managing complex, high-dimensional data and are proficient in handling feature interactions with complexity. This makes them particularly suitable for studies focused on detailed and complex aspects of multimorbidity patterns. Utilizing a novel evolutionary machine learning approach, we illustrate the ability to derive meaningful results even from rare events. Our model’s successful application in uncovering prevalent morbidity patterns linked to COVID-19 outcomes underscores its potential to yield valuable insights across diverse data sets, particularly where data sparsity poses challenges. While acknowledging its computational demands, we emphasize the model’s readiness and adaptability for analyzing complex medical data, highlighting its robustness as a powerful tool in medical research.

We identified prevalent morbidity patterns from the evolved data set, focusing on multimorbidity combinations or feature subsets closely associated with the outcome. This research targets clinically significant patterns directly. Utilizing an evolutionary algorithm to identify these combinations ensures the analysis is grounded in a robust, data-driven process. Analyzing the frequency of these subsets provides a measure of their prevalence and significance in the studied population. This step helps validate the relevance of the identified combinations, ensuring that the observed patterns are not random but indicative of common trends in patient data. Focusing on the most prevalent combinations, the study aims to yield findings with practical implications for health care providers. These findings can inform clinical decision-making by helping practitioners identify patients at higher risk due to specific multimorbidity patterns, enabling them to tailor treatment approaches accordingly.

Multimorbidity features such as older age combined with specific ATC codes (N03AX and R03BA) were frequently observed in outcome-related bins, particularly among middle-aged females. Likewise, during the analysis of SHAP values in cohort 1, it was noted that the use of inhaled corticosteroid medication for asthma (R03BA) had a significantly positive impact on the likelihood of hospitalization. This observation aligns with findings from the Open SAFELY study, which identified asthma as a significant risk factor for mortality in patients with COVID-19. Specifically, it highlighted that individuals using inhaled corticosteroids face the highest risk in this context [38].

The ATC N03AX group encompasses various antiepileptic medications used in treating bipolar disorder, epilepsy, migraine, and sometimes schizophrenia. Individuals with severe mental illnesses have shown a slightly higher risk of severe clinical outcomes from COVID-19 compared with those without prior mental health conditions [39]. Also, there have been reports linking the use of antiepileptic medications with vitamin D deficiency [40]. In our study, the presence of A11CC (vitamin D and analogs) in the multimorbidity history makes middle-aged females more vulnerable to hospitalization. Conversely, for older-age females, the presence of this feature is associated with smaller SHAP values, indicating that its presence in their history is protective against hospitalization.

In a multimorbidity study of hospitalized patients with COVID-19 [41], the ATC group most closely associated with prolonged hospital stays is M04AA, which includes preparations inhibiting uric acid production. In our study, among older-age females, the combinations of M04AA and NO3AX were notably frequent. M04AA also featured prominently in middle-aged males, while G04CA (alpha-adrenoreceptor antagonists), used for benign prostatic hypertrophy, was notable among older-age males. Research indicates that male COVID-19 cohorts experience more unfavorable clinical outcomes compared with females [42,43]. Specifically, while patients with cancer are at an increased risk of SARS-CoV-2 infection, individuals undergoing androgen-deprivation therapy for prostate cancer appear to have some level of protection against the infection [43].

### Strength and Limitations

Each row in the data set represents a comprehensive aggregation of each patient’s multimorbidity history over a 5-year period, including all relevant instances of diseases and conditions. This approach ensures a holistic view of each patient’s health status. To minimize subjectivity in the selection process, the criteria for including health records in the data set are consistent and objective. The aggregation process is governed by standardized criteria, uniformly applied across all patients. Also, aggregating multiple health records into a single patient instance helps mitigate bias that could arise from selectively choosing one entry over another.

In many clinical scenarios, understanding the implications of false positives and false negatives is a requisite beyond just disease probabilities. Although metrics such as Pietra and sBrier [44] and the average deviation about the probability threshold (ADAPT) index [45] are valuable, especially when patients seek to understand disease probabilities, we believe that traditional metrics such as AUC, accuracy, precision, recall, and *F*_1_-score, along with a confusion matrix, offer a comprehensive evaluation of the prediction models in this study. The use of a confusion matrix as an evaluation tool enables us to customize model assessment to reflect different clinical priorities, which is particularly relevant when the prediction model informs treatment plans or risk assessments [46].

Evolutionary algorithms inherently favor the best performing choices available, despite their stochastic nature. These biases contribute to their improved performance. Each evolutionary cycle involves evaluating bin fitness and performing genetic operations to identify the best performing group of features. In this study, the evolutionary algorithm is used not only for feature selection in sparse data but also to indirectly assess epistatic associations between features in each evolutionary cycle. Multimorbidity features are grouped into bins and scored based on a deep learning classifier’s predictive ability for the outcome. The features within bins are regrouped iteratively after each evolutionary cycle.

Many studies using machine learning to investigate multimorbidity patterns focus on handling sparse data sets by either removing sparsity-generating features or merging feature categories to reduce sparsity. However, these methods often result in information loss and less precise interpretation of multimorbidity features [19]. Instead of relying solely on a sequential deep learning model, we aggregated all evolved bins to create a new data set. This allowed us to analyze the evolutionarily evolved bins and identify frequent multimorbidity features and combinations.

Analyzing all possible combinations of multimorbidity features in a data set can be computationally expensive, and many irrelevant combinations may not warrant further analysis. To address this, we applied an evolutionary algorithm to extract meaningful combinations, prioritizing even less prevalent features. Consequently, our focus shifted to investigating only the most common multimorbidity features found in the top bins.

### Conclusions

When combined with other multimorbidity features, we identified associations with the outcome even for less prevalent medical conditions. Discovering hidden interconnections among different multimorbidity features opens new research pathways for studying multidimensional medical conditions in combination.

Using an innovative evolutionary machine learning approach, we identified prevalent morbidity patterns linked to hospitalization risk, especially among specific age and gender cohorts. Our findings highlight the adaptability of this methodology, demonstrating its ability to yield significant insights even in scenarios involving rare events. In addition to this, we repurposed administrative data for multimorbidity analysis, offering a novel path for public health research. This approach has the potential to influence future studies and interventions, encompassing areas such as polypharmacy and long COVID-19 research. By deepening our understanding of COVID-19 dynamics, this study emphasizes the broader utility of such methodologies in shaping effective public health strategies and interventions.

## Appendix

Multimedia Appendix 1 One-proportion *z*-test results.

Multimedia Appendix 2 Performance evaluation of other machine learning algorithms.

Multimedia Appendix 3 Outcome association of the feature and the support.

Multimedia Appendix 4 Most prevalent multimorbidity feature combinations in evolved bins.

Multimedia Appendix 5 The code used to derive the study results.

## Abbreviations

AdaGrad: Adaptive Gradient Algorithm
ADAPT: average deviation about the probability threshold
ATC: Anatomical Therapeutic Chemical
AUC: area under the curve
ICD-9-CM: 9th International Classification of Diseases-Clinical Modification
ISTAT: Istituto Nazionale di Statistica
PLS: Piedmont Longitudinal Study
PSN: Programma Statistico Nazionale
SHAP: Shapley Additive Explanations
SISTAN: National Statistical System

## Data Availability

The data sets used in this study are not publicly available. As a result of ethical committee restrictions, raw data cannot be publicly or freely shared to ensure the privacy and protection of individual-level data. However, researchers may request access to aggregated data by contacting the corresponding author (DB) through a reasonable inquiry. The code utilized to derive the results in this study is accessible in Multimedia Appendix 5.
