# Supplementary material for: A Multimorbidity Analysis of Hospitalized Patients With COVID-19 in Northwest Italy: Longitudinal Study Using Evolutionary Machine Learning and Health Administrative Data
Source: JMIR Public Health Surveill. 2024 Jul 18;10:e52353. doi: 10.2196/52353 (PMC11294776; doi:10.2196/52353)
Supplement: Multimedia Appendix 1 [file publichealth_v10i1e52353_app1.pdf]

One Proportion *z*-test Results

If P value <= 0.05, the features are eliminated from the sampled dataset.

Cohort 1

Features	*z* score	*P* value	Prevalence of the feature in raw data (%)	Prevalence of the feature in sampled data (%)
ATC J01CR	-1.92	.06	47.2	44.48
ATC H02AB	-1.68	.09	43.67	41.31
Age > 53	-0.14	.89	41.34	41.15
ATC J01FA	-1.25	.21	36.77	35.06
ATC A02BC	-2.57	.01	32.39	29.06
ATC J01MA	-1.78	.07	29.28	27.03
ATC M01AB	-1.78	.07	26.11	23.94
ATC A11CC	-1.34	.18	24.66	23.05
ATC M01AE	-2.65	.008	21.44	18.51
ATC J01DD	-0.13	.9	21.33	21.19
ATC R03BA	-1.04	.3	16.57	15.5
ATC J01XX	-1.48	.14	15.68	14.2
ATC J01CA	-0.76	.45	14.88	14.12
ATC N06AB	-0.77	.44	13.8	13.07
ATC B03AA	-0.33	.74	11.99	11.69
ATC H03AA	-1.1	.27	11.77	10.8
ATC J02AC	-0.41	.68	11.08	10.71
ATC A02AD	-1.66	.10	10.97	9.58
ATC N02BE	-1.47	.14	10.72	9.5
ATC C07AB	-0.57	.57	10.14	9.66
ATC A07AA	-0.29	.77	9.98	9.74
ATC R03AK	-1.3	.19	9.56	8.52
ATC M01AH	-0.4	.69	9.34	9.01
ATC C09AA	-1.73	.08	9.02	7.71
ATC A02BX	-0.8	.43	8.06	7.47
ATC N02AX	-1.06	.29	7.66	6.9
ATC N02AA	-0.59	.55	7.33	6.9
ATC R06AX	-0.45	.65	7.06	6.74
ATC B03BB	-0.74	.46	6.68	6.17
ATC N06AX	-0.02	.99	6.34	6.33
ATC C08CA	-0.71	.48	6.32	5.84
ATC N03AX	-0.96	.34	6.23	5.6
ATC C10AA	-1.64	.10	5.96	4.95
ATC C09CA	-0.54	.59	5.79	5.44
ATC B01AB	-0.76	.44	5.76	5.28
ATC N02CC	-0.59	.56	5.65	5.28
ATC R06AE	-1.6	.11	5.14	4.22
ATC R03AC	-1.03	.3	5.07	4.46
ATC A12AX	-0.63	.53	4.67	4.3
ATC C03CA	0.87	.39	4.65	5.19
ATC J05AB	-1.01	.31	4.62	4.06

ATC M01AX	-1.24	.22	4.58	3.9
ATC B01AC	-0.18	.85	4.24	4.14
ATC C09DA	-1.02	.31	4.11	3.57
ATC M01AC	-2.63	.009	3.89	2.68
ATC G03DB	-0.91	.36	3.71	3.25
ATC B02AA	-0.13	.9	3.64	3.57
ATC C09BA	-0.12	.91	3.31	3.25
ATC G03CA	-1.12	.26	3.28	2.76
ATC A10BA	-0.07	.94	3.28	3.25
ATC N06AA	-1.69	.09	3.08	2.35
ATC A02BA	0.59	.55	2.95	3.25
ATC P01AB	-0.21	.83	2.86	2.76
ICD 621	0.92	.36	2.26	2.68
ATC J01EE	0.46	.65	2.23	2.44
ATC G03AA	0.51	.61	2.21	2.44
ATC J01AA	0.38	.7	2.19	2.35
ATC A03FA	-1.01	.31	2.17	1.79
ATC C03EA	0.54	.59	2.12	2.35
ATC A07EC	-0.06	.95	2.05	2.03
ATC D05AX	0.51	.61	2.05	2.27
ATC C09BB	-1.21	.23	1.97	1.54
ICD 218	-1.56	.12	1.9	1.38
ATC J01DC	0.99	.32	1.85	2.27
ICD 727	-0.12	.9	1.83	1.79
ATC A05AA	0.21	.84	1.79	1.87
ICD 574	-0.63	.53	1.68	1.46
ATC N03AG	-0.12	.9	1.59	1.54
ATC M04AA	0.56	.57	1.5	1.7
ATC A12AA	-0.82	.41	1.47	1.22
ATC C02CA	-0.54	.59	1.47	1.3
ATC C07AA	0.69	.49	1.45	1.7
ATC R03DC	-2.24	.02	1.38	0.81
ATC B03BA	-0.2	.84	1.36	1.3
ATC R03AL	0.99	.32	1.34	1.7
ICD 454	0.19	.85	1.32	1.38
ATC S01ED	-0.75	.45	1.18	0.97
ICD 735	-0.44	.66	1.18	1.06
ATC C07BB	-0.29	.77	1.14	1.06
ICD V58	1.41	.16	1.12	1.62
ATC C03DA	-0.75	.45	1.09	0.89
ATC C03BA	1.14	.26	1.07	1.46
ATC P01BA	-0.93	.35	1.05	0.81
ATC N02BA	0.36	.72	1.03	1.14
ICD 174	0.17	.86	1.01	1.06
ATC R03BB	-1.46	.15	0.98	0.65
ATC C10AX	-0.25	.8	0.96	0.89
ATC N03AF	-0.49	.62	0.94	0.81
ATC M05BA	-1.16	.24	0.92	0.65

ATC A12BA	0.48	.63	0.92	1.06
ATC N03AE	0.08	.94	0.87	0.89
ICD 278	0.08	.94	0.87	0.89
ATC L01BA	0.61	.54	0.8	0.97
ICD 717	0.33	.74	0.8	0.89
ATC A07EA	0.33	.74	0.8	0.89
ATC C09BX	-1.49	.14	0.78	0.49
ATC C09DB	-2.68	.007	0.76	0.32
ICD 338	-0.29	.77	0.71	0.65
ICD 626	-1.04	.3	0.69	0.49
ATC A10AB	-0.19	.85	0.69	0.65
ICD 726	-0.19	.85	0.69	0.65
ATC A10BB	0.25	.8	0.67	0.73
ATC N01BB	0.91	.36	0.65	0.89
ATC N05AD	0.34	.73	0.65	0.73
ICD 715	-1.99	.05	0.65	0.32
ATC C10AB	0.34	.73	0.65	0.73
ATC N02AB	0.73	.47	0.63	0.81
ATC N02AJ	-0.7	.49	0.63	0.49
ATC S01EE	-1.72	.09	0.6	0.32
ATC C03AA	-0.47	.64	0.58	0.49
ATC S01EC	-0.06	.95	0.58	0.57
ICD 473	-0.25	.8	0.54	0.49
ICD 455	-1.3	.19	0.54	0.32
ATC N04AA	-0.6	.55	0.51	0.41
ATC C10BA	0.89	.37	0.51	0.73
ICD 553	-0.47	.64	0.49	0.41
ICD V54	0.09	.93	0.47	0.49
ATC C03EB	-0.35	.73	0.47	0.41
ICD 296	1.08	.28	0.47	0.73
ATC N03AA	-2.48	.01	0.45	0.16
ATC C07AG	0.57	.57	0.45	0.57
ATC B01AA	0.89	.38	0.45	0.65
ATC N05AA	-0.75	.45	0.45	0.32
ICD V64	0.67	.5	0.42	0.57
ATC N05AH	0.43	.67	0.4	0.49
ATC R03DA	0.02	.98	0.4	0.41
ICD 592	0.43	.67	0.4	0.49
ATC B05BB	-0.97	.33	0.38	0.24
ICD 241	-1.51	.13	0.34	0.16
ICD 996	-0.65	.51	0.34	0.24
ICD 618	0.39	.7	0.34	0.41
ATC C01BC	-0.49	.62	0.31	0.24
ICD 038	1.47	.14	0.31	0.65
ICD 812	0.88	.38	0.31	0.49
ICD 354	-1.12	.26	0.29	0.16
ATC C02AC	0.64	.52	0.29	0.41
ICD 478	-0.33	.74	0.29	0.24

ICD V53	-0.17	.86	0.27	0.24
ICD 301	0.35	.73	0.27	0.32
ICD 780	0.35	.73	0.27	0.32
ICD 295	-0.02	.99	0.25	0.24
ICD 298	-0.53	.59	0.22	0.16
ICD 518	1.33	.18	0.22	0.49
ATC C01DA	-0.53	.59	0.22	0.16
ICD 434	-1.75	.08	0.22	0.08
ICD 562	-0.53	.59	0.22	0.16
ICD 998	0.14	.89	0.22	0.24
ICD 599	-0.34	.74	0.2	0.16
ICD 585	1.13	.26	0.2	0.41
ICD V43	-1.48	.14	0.2	0.08
ICD 427	0.3	.76	0.2	0.24
ICD 722	0.3	.76	0.2	0.24
ICD 786	-1.2	.23	0.18	0.08
ICD 820	-0.14	.89	0.18	0.16
ICD 550	-0.14	.89	0.18	0.16
ICD V56	1.25	.21	0.18	0.41
ICD 410	-0.14	.89	0.18	0.16
ICD 470	0.46	.64	0.18	0.24
ICD V57	0.46	.64	0.18	0.24
ICD 560	-0.93	.35	0.16	0.08
ICD 482	0.78	.44	0.13	0.24
ICD 813	0.25	.8	0.13	0.16
ICD 486	0.44	.66	0.11	0.16
ICD 041	0.44	.66	0.11	0.16
ICD 211	0.44	.66	0.11	0.16
ICD 162	-0.1	.92	0.09	0.08
ICD 437	-0.1	.92	0.09	0.08
ICD 424	1.1	.27	0.09	0.24
ICD 995	0.64	.52	0.09	0.16
ICD V71	-0.1	.92	0.09	0.08
ICD 571	-0.1	.92	0.09	0.08
ICD 728	0.17	.86	0.07	0.08
ICD 428	0.17	.86	0.07	0.08
ATC C01BD	1.26	.21	0.07	0.24
ICD 188	0.17	.86	0.07	0.08
ICD 250	0.83	.41	0.07	0.16
ICD 438	0.45	.65	0.04	0.08
ICD 366	0.45	.65	0.04	0.08
ICD 411	0.45	.65	0.04	0.08
ICD 440	0.73	.47	0.02	0.08
ICD 153	0.73	.47	0.02	0.08

Removed features: ATC A02BC, ATC M01AE, ATC M01AC, ATC R03DC, ATC C09DB, ICD 715, ATC N03AA

Cohort 2

Features	*z* score	*P* value	Prevalance of the feature in raw data (%)	Prevalance of the feature in sampled data (%)
Age > 53	-0.72	.47	45.45	44.69
ATC J01CR	-1.07	.28	41.45	40.33
ATC H02AB	-1.25	.21	34.77	33.51
ATC J01FA	-0.76	.45	29.43	28.7
ATC J01MA	-1.76	.08	27.43	25.79
ATC A02BC	-3.06	.002	26.91	24.11
ATC M01AB	-1.73	.08	19.63	18.21
ATC J01DD	-1.34	.18	16.89	15.85
ATC M01AE	-0.78	.43	16.23	15.62
ATC R03BA	-1.33	.18	13.94	12.99
ATC C09AA	-1.25	.21	12.86	11.99
ATC J01CA	-0.67	.51	11.94	11.49
ATC C10AA	-2.93	.003	10.92	9.13
ATC A07AA	-1.61	.11	10.78	9.76
ATC C07AB	-2.24	.02	10.75	9.36
ATC C09CA	-0.82	.41	8.89	8.4
ATC N06AB	-0.51	.61	8.89	8.58
ATC C08CA	-1.46	.14	8.22	7.4
ATC R03AK	-0.82	.41	8.18	7.72
ATC G04CA	-1.96	.05	8.01	6.95
ATC B01AC	-2.68	.007	7.55	6.18
ATC A02AD	-1.61	.11	7.48	6.63
ATC A11CC	-0.74	.46	7.02	6.63
ATC N02BE	-0.59	.56	6.71	6.4
ATC J02AC	-0.9	.37	6.64	6.18
ATC A02BX	-0.69	.49	6.25	5.9
ATC C09DA	-1.16	.25	5.87	5.31
ATC M04AA	-1.26	.21	5.73	5.13
ATC B01AB	-0.52	.6	5.66	5.4
ATC N03AX	-0.97	.33	5.44	5
ATC M01AH	-1.45	.15	5.23	4.59
ATC N02AA	-1.26	.21	5.2	4.63
ATC R06AX	-0.66	.51	5.16	4.86
ATC A10BA	-1.8	.07	5.09	4.31
ATC N02AX	-1.07	.29	4.78	4.31
ATC N06AX	-1.22	.22	4.6	4.09
ATC C03CA	-1.05	.29	4.53	4.09
ATC B03BB	-1.31	.19	4.5	3.95
ATC J01XX	-0.69	.49	4.43	4.13
ATC R03AC	-0.11	.91	4.18	4.13
ATC C09BA	-0.81	.42	4.14	3.81
ATC M01AX	-1.57	.12	3.86	3.27

ATC C02CA	-0.95	.34	3.58	3.22
ATC C09BB	-0.39	.7	3.23	3.09
ATC B03AA	-1.12	.26	3.16	2.77
ATC R06AE	-1.12	.26	3.16	2.77
ATC J05AB	-0.52	.61	3.09	2.91
ATC J01EE	-0.85	.39	3.02	2.72
ATC H03AA	-1.15	.25	2.88	2.5
ATC N03AG	-0.94	.35	2.81	2.5
ATC C10AX	-0.8	.43	2.81	2.54
ATC A07EC	-0.59	.56	2.74	2.54
ATC D05AX	-0.59	.56	2.6	2.41
ATC M01AC	-0.23	.82	2.53	2.45
ATC A07EA	-1.22	.22	2.21	1.86
ATC C10AB	-0.25	.8	2.07	2
ATC N02BA	-1.43	.15	2.07	1.68
ICD 550	0.62	.53	2.07	2.27
ATC A10AB	-0.57	.57	1.93	1.77
ATC C09DB	-0.74	.46	1.93	1.73
ATC A02BA	-1.15	.25	1.9	1.59
ATC N03AE	-0.94	.35	1.79	1.54
ATC N02CC	0.25	.81	1.79	1.86
ATC A10BB	-1.29	.2	1.58	1.27
ATC P01AB	-0.14	.89	1.58	1.54
ATC C03DA	-1	.32	1.51	1.27
ICD 717	-0.13	.9	1.44	1.41
ATC S01ED	-0.22	.83	1.37	1.32
ATC J01AA	-0.46	.64	1.33	1.23
ATC B03BA	-2.11	.03	1.33	0.91
ATC A05AA	0.03	.98	1.26	1.27
ATC R03AL	-0.36	.72	1.26	1.18
ATC N05AD	-0.36	.72	1.26	1.18
ATC N03AF	-0.21	.83	1.23	1.18
ATC N05AH	-0.06	.95	1.19	1.18
ATC G04CB	-0.76	.45	1.16	1
ATC N04AA	-0.53	.6	1.16	1.04
ATC N06AA	-0.53	.6	1.16	1.04
ATC J01DC	-0.31	.75	1.16	1.09
ICD 574	-0.89	.37	1.09	0.91
ICD V58	-0.2	.84	1.09	1.04
ICD 727	-0.42	.67	1.09	1
ATC A12AX	-0.2	.84	1.09	1.04
ATC C01DA	-1.41	.16	1.09	0.82
ATC B01AA	-0.97	.33	1.05	0.86
ATC A03FA	-0.26	.8	1.05	1
ATC R03BB	-0.97	.33	1.05	0.86
ICD 592	-0.48	.63	1.05	0.95
ATC C09BX	-0.31	.75	1.02	0.95
ATC N05AA	-0.37	.71	0.98	0.91

ATC B02AA	-1.13	.26	0.98	0.77
ICD 454	0.28	.78	0.98	1.04
ATC R03DC	-0.95	.34	0.95	0.77
ICD 410	-1.64	.10	0.91	0.64
ATC A12AA	0.15	.88	0.88	0.91
ATC C03BA	-0.57	.57	0.88	0.77
ICD 553	-0.13	.89	0.84	0.82
ATC A12BA	0.1	.92	0.84	0.86
ATC C10BA	-0.92	.36	0.84	0.68
ATC C07AG	-0.19	.85	0.81	0.77
ATC C03EA	-0.45	.65	0.81	0.73
ATC C07BB	-0.72	.47	0.81	0.68
ATC N03AA	-0.81	.42	0.77	0.64
ATC S01EE	-0.52	.6	0.77	0.68
ICD 715	0.42	.68	0.74	0.82
ICD 038	-0.78	.44	0.67	0.54
ATC C02AC	-0.26	.8	0.63	0.59
ATC R03DA	-0.88	.38	0.63	0.5
ATC B05BB	-0.88	.38	0.63	0.5
ICD 518	-0.26	.8	0.63	0.59
ICD 455	-0.11	.91	0.56	0.54
ICD 473	-0.11	.91	0.56	0.54
ATC N02AB	-0.75	.45	0.56	0.45
ICD 413	-0.11	.91	0.56	0.54
ICD 470	-0.42	.68	0.56	0.5
ATC L01BA	-0.75	.45	0.56	0.45
ATC C07AA	-0.51	.61	0.53	0.45
ATC N02AJ	-0.18	.86	0.53	0.5
ICD V53	0.12	.91	0.53	0.54
ATC C03AA	-1.28	.2	0.53	0.36
ICD 600	0.05	.96	0.49	0.5
ICD 301	-1.45	.15	0.49	0.32
ATC C03EB	0.34	.73	0.49	0.54
ATC S01EC	-0.02	.99	0.46	0.45
ATC C01BC	-1.16	.25	0.46	0.32
ICD 585	0.29	.78	0.46	0.5
ATC N01BB	0.52	.6	0.42	0.5
ICD 298	0.23	.82	0.42	0.45
ICD 214	0.52	.6	0.42	0.5
ICD 278	0.23	.82	0.42	0.45
ICD 996	0.16	.87	0.39	0.41
ICD 995	-1.03	.31	0.39	0.27
ICD 427	-0.57	.57	0.39	0.32
ICD 722	-0.18	.86	0.39	0.36
ICD 434	-1.03	.31	0.39	0.27
ICD 482	0.09	.93	0.35	0.36
ICD 726	-0.28	.78	0.35	0.32
ICD 295	0.42	.67	0.35	0.41

ICD V43	0.09	.93	0.35	0.36
ICD 414	-0.71	.48	0.35	0.27
ICD 438	-1.22	.22	0.35	0.23
ICD 571	-0.71	.48	0.35	0.27
ICD 478	-0.88	.38	0.32	0.23
ICD 786	-0.39	.69	0.32	0.27
ICD V54	0.01	.99	0.32	0.32
ICD 431	-0.39	.69	0.32	0.27
ICD 486	-0.39	.69	0.32	0.27
ATC C01BD	-1.09	.27	0.28	0.18
ICD 560	-0.08	.94	0.28	0.27
ICD 296	-0.53	.59	0.28	0.23
ICD 411	-1.84	.07	0.28	0.14
ICD 728	-0.08	.94	0.28	0.27
ICD 415	-1.84	.07	0.28	0.14
ICD 338	-0.08	.94	0.28	0.27
ICD 428	0.31	.76	0.28	0.32
ICD V64	-1.09	.27	0.28	0.18
ICD V57	-1.84	.07	0.28	0.14
ICD V71	-0.19	.85	0.25	0.23
ICD 211	-0.19	.85	0.25	0.23
ICD 780	-0.19	.85	0.25	0.23
ICD 424	-0.32	.75	0.21	0.18
ICD V56	0.56	.58	0.21	0.27
ICD 241	0.16	.87	0.21	0.23
ICD 173	0.16	.87	0.21	0.23
ATC P01BA	-0.95	.34	0.21	0.14
ICD 812	0.07	.95	0.18	0.18
ICD 250	0.07	.95	0.18	0.18
ICD 041	-0.5	.62	0.18	0.14
ICD 437	-0.5	.62	0.18	0.14
ICD 998	0.51	.61	0.18	0.23
ICD 354	-0.5	.62	0.18	0.14
ICD 440	-0.05	.96	0.14	0.14
ICD 366	-0.05	.96	0.14	0.14
ICD 735	0.45	.65	0.14	0.18
ICD 188	0.45	.65	0.14	0.18
ICD 562	0.45	.65	0.14	0.18
ICD 813	-0.05	.96	0.14	0.14
ICD 153	-0.23	.82	0.11	0.09
ICD 185	-0.23	.82	0.11	0.09
ICD 584	0.39	.69	0.11	0.14
ATC M05BA	-0.23	.82	0.11	0.09
ICD 599	-0.23	.82	0.11	0.09
ICD 820	-1.32	.19	0.11	0.05
ICD 276	-0.55	.58	0.07	0.05
ICD 162	-0.55	.58	0.07	0.05
ATC G03DB	0.23	.82	0.04	0.05

ICD 331	0.23	.82	0.04	0.05

Removed features: ATC A02BC, ATC C10AA, ATC C07AB, ATC B01AC, ATC B03BA

Cohort 3

**Features**	***z* score**	***P* value**	**Prevalance of the feature in raw data (%)**	**Prevalance of the feature in sampled data (%)**
ATC J01CR	0.16	.87	54.01	54.2
ATC A02BC	-0.13	.89	49.47	49.32
ATC H02AB	-0.09	.93	47.69	47.59
ATC A11CC	0.13	.9	45.18	45.33
ATC J01MA	-0.19	.85	41.4	41.19
ATC J01FA	-0.32	.75	38.6	38.25
Age > 68	1.42	.15	37.03	38.61
ATC M01AB	-0.05	.96	33.16	33.11
ATC M01AE	1.1	.27	30.62	31.79
ATC J01DD	-0.01	.99	29.13	29.12
ATC C10AA	0.04	.97	26.24	26.29
ATC C07AB	0.18	.86	24.59	24.76
ATC R03BA	-0.91	.36	23.27	22.4
ATC N06AB	0.18	.85	22.12	22.3
ATC J01XX	0.03	.98	21.49	21.51
ATC B01AC	-0.16	.87	21.19	21.04
ATC C03CA	-0.1	.92	20.08	19.99
ATC C09AA	0.09	.93	19.49	19.57
ATC M01AH	0.54	.59	17.83	18.31
ATC A07AA	0.12	.91	17.58	17.68
ATC J01CA	-0.63	.53	17.54	17
ATC H03AA	-0.73	.47	17.2	16.58
ATC N02BE	0.22	.83	17.07	17.26
ATC C08CA	0.43	.67	15.8	16.16
ATC A02AD	-0.69	.49	15.63	15.06
ATC R03AK	-0.19	.85	15.37	15.22
ATC C09CA	0.48	.63	15.03	15.42
ATC C09DA	0.72	.47	14.31	14.9
ATC N02AA	-0.03	.98	13.93	13.9
ATC N06AX	0.1	.92	13.04	13.12
ATC N02AX	0.23	.82	12.78	12.96
ATC A12AX	-0.77	.44	12.7	12.12
ATC A02BX	-0.74	.46	12.57	12.01
ATC B01AB	0.53	.59	12.19	12.59
ATC N03AX	-0.14	.89	11.8	11.7
ATC A10BA	0.18	.86	11.25	11.39
ATC B03BB	-1.06	.29	9.94	9.23
ATC M04AA	-0.26	.8	8.66	8.5
ATC M01AX	-0.26	.8	8.66	8.5
ATC C09BA	-0.12	.9	8.58	8.5
ATC J02AC	0.54	.59	8.15	8.5

ATC B03AA	-0.73	.47	7.94	7.5
ATC R03AC	-0.05	.96	7.22	7.19
ATC M01AC	0.51	.61	6.84	7.14
ATC J05AB	-0.64	.52	6.11	5.77
ATC R06AE	0.05	.96	5.9	5.93
ATC N02BA	0.05	.96	5.9	5.93
ATC R06AX	-0.67	.5	5.86	5.51
ATC G03CA	-0.18	.86	5.39	5.3
ATC C02CA	0.5	.62	5.35	5.61
ATC M05BA	0.08	.93	5.31	5.35
ATC R03BB	0.07	.94	5.05	5.09
ATC J01EE	0.16	.88	5.01	5.09
ATC C03EA	0.91	.36	4.88	5.35
ATC A12AA	-0.09	.93	4.71	4.67
ATC C03DA	0.06	.95	4.59	4.62
ATC A07EC	0.04	.96	4.54	4.56
ATC A02BA	-0.83	.4	4.42	4.04
ATC A10AB	0.01	.99	4.25	4.25
ATC C09BB	0.81	.42	4.08	4.46
ATC C10AX	-0.01	.99	3.99	3.99
ATC A12BA	-0.03	.97	3.95	3.93
ATC N06AA	0.3	.77	3.91	4.04
ATC A10BB	0	1.0	3.78	3.78
ATC S01ED	0.56	.58	3.74	3.99
ATC D05AX	-0.82	.41	3.69	3.36
ATC A05AA	0	1.0	3.57	3.57
ATC B01AA	0.38	.7	3.35	3.52
ATC A03FA	-0.15	.88	3.31	3.25
ATC N03AG	0.36	.72	3.31	3.46
ICD 715	0.09	.93	3.27	3.31
ATC C03EB	0.42	.67	3.18	3.36
ATC B03BA	-0.88	.38	3.06	2.73
ATC N05AD	-0.45	.65	3.06	2.89
ATC C03BA	0.71	.48	3.01	3.31
ATC R03AL	0.22	.83	2.8	2.89
ATC N02CC	-0.09	.93	2.76	2.73
ICD V58	-0.4	.69	2.72	2.57
ATC C07BB	-0.03	.98	2.63	2.62
ATC P01AB	-0.86	.39	2.55	2.26
ATC C10BA	-0.23	.82	2.55	2.47
ATC C01DA	0.18	.86	2.51	2.57
ATC J01DC	0.46	.64	2.51	2.68
ATC N02AJ	0.32	.75	2.51	2.62
ICD 518	0.07	.94	2.34	2.36
ATC C07AA	-0.27	.79	2.29	2.2
ATC N02AB	0.61	.54	2.25	2.47
ATC N03AE	0.11	.91	2.17	2.2
ATC P01BA	-0.27	.79	2.08	1.99

ATC C09DB	0.64	.52	2.04	2.26
ATC B05BB	-0.64	.52	1.87	1.68
ICD V43	0.23	.82	1.87	1.94
ATC L01BA	-0.1	.92	1.87	1.84
ICD 574	0.17	.86	1.78	1.84
ICD 727	0.34	.74	1.78	1.89
ATC S01EE	0.14	.89	1.74	1.78
ATC R03DA	-0.4	.69	1.74	1.63
ATC C10AB	0.45	.65	1.7	1.84
ATC S01EC	0.45	.65	1.7	1.84
ICD 038	0.45	.65	1.7	1.84
ATC N03AA	-0.29	.77	1.66	1.57
ATC B02AA	-0.33	.74	1.61	1.52
ATC N03AF	-0.52	.6	1.61	1.47
ATC R03DC	-1	.32	1.57	1.31
ATC N04AA	0.19	.85	1.57	1.63
ATC N05AA	0.19	.85	1.57	1.63
ATC C07AG	-0.37	.71	1.57	1.47
ATC N05AH	0.34	.74	1.53	1.63
ATC C01BC	-0.67	.5	1.49	1.31
ATC J01AA	0.65	.51	1.49	1.68
ICD 338	0.09	.93	1.44	1.47
ICD 621	0.09	.93	1.44	1.47
ATC N01BB	-0.3	.76	1.44	1.36
ATC C03AA	0.94	.35	1.4	1.68
ICD 296	-0.34	.73	1.4	1.31
ICD 820	-0.61	.54	1.36	1.21
ICD 996	-0.22	.82	1.32	1.26
ICD 410	-0.06	.95	1.27	1.26
ATC C02AC	-0.06	.95	1.27	1.26
ICD 174	-0.06	.95	1.27	1.26
ICD 735	0.14	.88	1.27	1.31
ICD 454	-0.14	.89	1.19	1.15
ICD 428	0.07	.94	1.19	1.21
ICD 427	0.03	.97	1.15	1.15
ATC C09BX	0.21	.84	1.1	1.15
ICD 618	0.21	.84	1.1	1.15
ICD 278	0.55	.58	1.02	1.15
ICD 585	-0.15	.88	0.98	0.94
ATC C01BD	0.09	.93	0.98	1
ATC A07EA	0.49	.62	0.93	1.05
ICD 486	-0.15	.88	0.76	0.73
ICD 295	-0.15	.88	0.76	0.73
ICD 366	0.32	.75	0.72	0.79
ICD 331	0.32	.75	0.72	0.79
ICD 482	0.32	.75	0.72	0.79
ICD 813	0.56	.57	0.72	0.84
ICD V56	-0.34	.73	0.64	0.58

ICD V53	0.24	.81	0.64	0.68
ICD 717	0.24	.81	0.64	0.68
ICD 812	0.19	.85	0.59	0.63
ICD 424	0.14	.88	0.55	0.58
ICD 411	-0.17	.87	0.55	0.52
ICD 995	0.14	.88	0.55	0.58
ICD 413	-1.33	.18	0.55	0.37
ICD 250	0.14	.88	0.55	0.58
ICD 786	-0.61	.54	0.51	0.42
ICD 241	0.09	.93	0.51	0.52
ICD 301	0.39	.7	0.51	0.58
ICD 414	0.09	.93	0.51	0.52
ICD 415	0.09	.93	0.51	0.52
ICD 434	-1.03	.3	0.51	0.37
ICD 728	-0.24	.81	0.51	0.47
ICD 550	0.03	.97	0.47	0.47
ICD 562	0.63	.53	0.47	0.58
ICD 437	0.63	.53	0.47	0.58
ICD 592	0.35	.73	0.47	0.52
ICD V57	0.03	.97	0.47	0.47
ICD V64	-0.32	.75	0.47	0.42
ICD 438	-0.03	.97	0.42	0.42
ICD V71	0.3	.76	0.42	0.47
ICD 571	-0.41	.68	0.42	0.37
ICD 726	0.3	.76	0.42	0.47
ICD 153	0.25	.8	0.38	0.42
ICD 211	0.25	.8	0.38	0.42
ICD 041	-1.02	.31	0.38	0.26
ICD 560	0.25	.8	0.38	0.42
ICD 173	0.25	.8	0.38	0.42
ICD 276	-0.11	.91	0.38	0.37
ICD V54	0.25	.8	0.38	0.42
ICD 599	-0.11	.91	0.38	0.37
ICD 780	-0.53	.6	0.38	0.31
ICD 218	-0.53	.6	0.38	0.31
ICD 478	-0.11	.91	0.38	0.37
ICD 188	-0.19	.85	0.34	0.31
ICD 584	0.54	.59	0.34	0.42
ICD 214	-0.3	.77	0.3	0.26
ICD 431	-0.83	.4	0.3	0.21
ICD 998	0.06	.95	0.25	0.26
ICD 455	-1.07	.28	0.25	0.16
ICD 553	0.06	.95	0.25	0.26
ICD 433	0.47	.64	0.25	0.31
ICD 440	-1.07	.28	0.25	0.16
ICD 722	0.06	.95	0.25	0.26
ICD 298	0.43	.67	0.21	0.26
ICD 473	-0.02	.98	0.21	0.21

ICD 162	0.38	.7	0.17	0.21
ICD 470	0.38	.7	0.17	0.21
ICD 626	0.33	.74	0.13	0.16
ICD 354	-1.43	.15	0.13	0.05
ATC G04CA	0.27	.79	0.08	0.1
ATC G03AA	-0.62	.54	0.08	0.05
ATC G03DB	0.19	.85	0.04	0.05

No features are removed from the sampled data.

Cohort 4

Features	z score	P value	Prevalance of the feature in raw data (%)	Prevalance of the feature in sampled data (%)
ATC A02BC	-0.31	.76	49.71	49.39
ATC J01CR	0.07	.95	48.39	48.46
Age > 68	-1.22	.22	41.78	40.53
ATC J01MA	0.44	.66	40.08	40.53
ATC H02AB	-0.11	.91	36.99	36.89
ATC J01FA	0.58	.56	32.85	33.42
ATC C10AA	-0.42	.67	31.73	31.32
ATC B01AC	-0.16	.87	31.34	31.18
ATC C07AB	-0.91	.36	28.84	27.98
ATC M01AB	-0.11	.92	27.07	26.97
ATC C09AA	-0.54	.59	26.81	26.32
ATC G04CA	-0.82	.41	26.49	25.75
ATC M01AE	0.82	.41	24.82	25.57
ATC J01DD	-0.09	.93	24.25	24.17
ATC C08CA	-0.66	.51	21.71	21.14
ATC C09CA	0.01	.99	17.53	17.54
ATC R03BA	0.67	.5	17.05	17.59
ATC C03CA	-0.51	.61	16.89	16.49
ATC A10BA	-0.49	.62	16.7	16.32
ATC M04AA	-0.22	.83	16.44	16.27
ATC A07AA	-0.17	.86	16.18	16.05
ATC J01CA	0.29	.77	14.26	14.47
ATC A11CC	-1.08	.28	13.97	13.2
ATC C09DA	-0.71	.48	13.62	13.11
ATC R03AK	0.26	.8	12.85	13.03
ATC N06AB	0.28	.78	12.04	12.24
ATC N02BE	-0.14	.89	11.14	11.05
ATC B01AB	1.21	.23	10.98	11.8
ATC M01AH	0.39	.69	10.53	10.79
ATC N03AX	-0.44	.66	10.28	10
ATC C09BA	-0.27	.78	10.08	9.91
ATC G04CB	-0.55	.58	9.99	9.65
ATC N02AA	-1.33	.18	9.92	9.12
ATC N02AX	-0.03	.97	9.54	9.52

ATC A02AD	0.1	.92	9.28	9.34
ATC B03BB	-0.44	.66	9.12	8.86
ATC N02BA	0.65	.52	8.99	9.39
ATC A02BX	-0.27	.79	8.8	8.64
ATC N06AX	0.38	.7	8.29	8.51
ATC C02CA	-0.57	.57	8.12	7.81
ATC M01AX	-0.07	.94	7.8	7.76
ATC B03AA	0.11	.91	6.87	6.93
ATC C10AX	-0.38	.7	6.78	6.58
ATC J01XX	-0.15	.88	6.74	6.67
ATC A10AB	-0.56	.57	6.65	6.36
ATC J02AC	0.29	.77	6.17	6.32
ATC C09BB	0.44	.66	6.01	6.23
ATC C03DA	-0.4	.69	5.94	5.75
ATC A10BB	0.07	.95	5.84	5.88
ATC R03AC	0.28	.78	5.78	5.92
ATC R03BB	-1.14	.26	5.43	4.91
ATC J01EE	-0.6	.55	5.14	4.87
ATC M01AC	-1.03	.3	5.01	4.56
ATC H03AA	-0.63	.53	4.66	4.39
ATC J05AB	-0.38	.71	4.59	4.43
ATC C01DA	-1.16	.25	4.56	4.08
ATC S01ED	0.59	.56	4.43	4.69
ATC B01AA	0.54	.59	4.37	4.61
ATC R06AX	-0.43	.66	4.3	4.12
ATC D05AX	0.86	.39	4.27	4.65
ICD 550	-0.08	.93	3.98	3.95
ATC A07EA	-0.27	.79	3.79	3.68
ATC A02BA	-0.53	.6	3.76	3.55
ATC R06AE	-1.2	.23	3.6	3.16
ATC A07EC	0.25	.81	3.5	3.6
ATC C10BA	0.38	.7	3.4	3.55
ATC C09DB	-0.58	.56	3.37	3.16
ATC N03AG	-0.17	.87	3.31	3.25
ATC C10AB	-0.99	.32	3.24	2.89
ATC R03AL	0.05	.96	3.05	3.07
ATC A12BA	-0.11	.91	2.89	2.85
ICD 600	-0.28	.78	2.86	2.76
ICD 518	-0.56	.57	2.73	2.54
ATC C09BX	-0.89	.37	2.7	2.41
ATC C03EA	0.41	.68	2.67	2.81
ATC A12AX	0.09	.93	2.6	2.63
ICD 410	0.35	.73	2.6	2.72
ATC A05AA	0.31	.75	2.57	2.68
ATC S01EE	0.44	.66	2.57	2.72
ATC C03BA	0.02	.98	2.54	2.54
ATC B03BA	0.75	.45	2.5	2.76
ICD 715	0.69	.49	2.44	2.68

ATC C07AG	-0.27	.79	2.41	2.32
ICD V58	-0.31	.76	2.38	2.28
ATC C03EB	-0.84	.4	2.31	2.06
ATC B02AA	0.54	.59	2.28	2.46
ICD 413	0.24	.81	2.25	2.32
ATC C07AA	0.61	.54	2.22	2.41
ATC C01BC	0.07	.95	2.22	2.24
ATC N06AA	0.03	.98	2.18	2.19
ATC C01BD	-0.01	.99	2.15	2.15
ICD 427	-0.82	.41	2.12	1.89
ATC N05AD	0.24	.81	2.12	2.19
ATC N03AE	0.48	.63	2.09	2.24
ATC J01DC	-0.17	.87	2.02	1.97
ICD 414	0.34	.73	1.96	2.06
ICD 038	-0.51	.61	1.89	1.75
ATC J01AA	-0.19	.85	1.89	1.84
ATC P01AB	0.12	.9	1.89	1.93
ATC A03FA	-0.39	.69	1.86	1.75
ICD 727	0.31	.76	1.8	1.89
ICD 428	-0.72	.47	1.77	1.58
ICD 574	0.53	.59	1.73	1.89
ATC N03AF	-0.96	.34	1.73	1.49
ATC C07BB	0.23	.82	1.73	1.8
ATC A12AA	-0.25	.8	1.73	1.67
ATC S01EC	0.19	.85	1.7	1.75
ICD 434	0.19	.85	1.7	1.75
ATC N02AJ	-1.35	.18	1.64	1.32
ATC C02AC	-0.69	.49	1.57	1.4
ATC N02AB	-0.2	.84	1.54	1.49
ICD 411	0.43	.67	1.51	1.62
ICD 996	-0.87	.38	1.48	1.27
ATC N05AA	-0.22	.83	1.41	1.36
ICD 188	-0.09	.93	1.38	1.36
ATC R03DA	-0.66	.51	1.38	1.23
ICD V43	-0.09	.93	1.38	1.36
ATC B05BB	-0.33	.74	1.35	1.27
ATC N03AA	-0.86	.39	1.28	1.1
ICD 440	0.27	.79	1.25	1.32
ATC N05AH	-0.78	.43	1.22	1.05
ATC N04AA	-0.86	.39	1.19	1.01
ICD 185	-1.53	.13	1.12	0.83
ATC L01BA	0.77	.44	1.09	1.27
ATC N02CC	0.22	.83	1.09	1.14
ICD V56	-0.53	.59	1.03	0.92
ICD 486	-0.61	.54	1	0.88
ICD 585	-0.85	.39	1	0.83
ICD 438	0.83	.4	1	1.18
ICD V57	-0.44	.66	0.96	0.88

ATC C03AA	0.22	.83	0.96	1.01
ICD 717	-0.11	.91	0.9	0.88
ICD 295	-0.35	.73	0.9	0.83
ICD 338	-0.42	.68	0.87	0.79
ICD 482	0.48	.63	0.87	0.96
ATC N01BB	-1.24	.22	0.87	0.66
ICD V53	0.22	.83	0.83	0.88
ATC R03DC	-0.58	.56	0.8	0.7
ICD 820	0.16	.87	0.8	0.83
ICD 296	-0.14	.89	0.77	0.75
ICD 780	0.33	.74	0.77	0.83
ICD 433	0.71	.48	0.74	0.88
ICD 431	-0.21	.83	0.74	0.7
ICD 331	0.04	.97	0.74	0.75
ICD 250	-1.19	.23	0.71	0.53
ICD 584	-0.37	.71	0.67	0.61
ICD 162	-1.32	.19	0.67	0.48
ICD 153	0.16	.88	0.67	0.7
ICD 276	-0.1	.92	0.67	0.66
ICD 599	-0.17	.86	0.64	0.61
ICD 592	-0.77	.44	0.64	0.53
ATC P01BA	0.34	.73	0.64	0.7
ICD 553	0.34	.73	0.64	0.7
ICD 728	-0.46	.65	0.64	0.57
ICD 722	0.34	.73	0.64	0.7
ICD 454	0.28	.78	0.61	0.66
ICD 571	0.75	.45	0.61	0.75
ICD 366	0.02	.98	0.61	0.61
ICD V64	0.28	.78	0.61	0.66
ICD 473	0.15	.88	0.55	0.57
ICD 726	-1.32	.19	0.51	0.35
ICD 786	0.36	.72	0.51	0.57
ICD V71	0.29	.77	0.48	0.53
ICD 455	0.56	.57	0.48	0.57
ICD 424	0.76	.44	0.45	0.57
ICD 278	-0.8	.43	0.45	0.35
ICD 560	-0.08	.94	0.45	0.44
ICD 995	0.15	.88	0.42	0.44
ICD 173	0.45	.65	0.42	0.48
ICD 437	0.15	.88	0.42	0.44
ICD 562	0.38	.7	0.39	0.44
ICD 041	0.07	.94	0.39	0.39
ATC M05BA	0.32	.75	0.35	0.39
ICD 211	-0.02	.98	0.35	0.35
ICD 298	-0.02	.98	0.35	0.35
ICD 354	-0.4	.69	0.35	0.31
ICD 998	-0.12	.9	0.32	0.31
ICD 478	0.24	.81	0.32	0.35

ICD 415	0.81	.42	0.29	0.39
ICD 214	0.5	.62	0.29	0.35
ICD 812	0.43	.67	0.26	0.31
ICD 813	-0.56	.57	0.22	0.18
ICD 735	-0.06	.96	0.22	0.22
ICD 470	-0.06	.96	0.22	0.22
ICD V54	-0.2	.84	0.19	0.18
ICD 301	0.17	.87	0.16	0.18
ICD 241	-0.14	.89	0.1	0.09
ICD 174	0.27	.79	0.03	0.04

No features are removed from the sampled data.
