# Supplementary material for: A Multimorbidity Analysis of Hospitalized Patients With COVID-19 in Northwest Italy: Longitudinal Study Using Evolutionary Machine Learning and Health Administrative Data
Source: JMIR Public Health Surveill. 2024 Jul 18;10:e52353. doi: 10.2196/52353 (PMC11294776; doi:10.2196/52353)
Supplement: Multimedia Appendix 2 [file publichealth_v10i1e52353_app2.pdf]

Performance Evaluation of other Machine Learning algorithms

Cohort 1: (5-fold CV Result)

Model	Accuracy	AUC	Recall	Prec.	F1	Kappa	MCC	TT (Sec)
Logistic Regression	0.7175	0.7634	0.6275	0.7588	0.6863	0.4333	0.4405	6.260
CatBoost Classifier	0.7193	0.7602	0.6037	0.7773	0.6792	0.4365	0.4480	5.214
Gradient Boosting Classifier	0.7148	0.7583	0.5963	0.7735	0.6725	0.4274	0.4395	0.286
Ada Boost Classifier	0.7039	0.7512	0.6294	0.7340	0.6770	0.4064	0.4112	0.132
Naive Bayes	0.6489	0.7502	0.3266	0.8968	0.4772	0.2905	0.3760	0.012
Random Forest Classifier	0.6878	0.7262	0.5945	0.7237	0.6524	0.3737	0.3802	0.246
Light Gradient Boosting Machine	0.6742	0.7241	0.5853	0.7044	0.6387	0.3465	0.3521	0.204
Extreme Gradient Boosting	0.6751	0.7216	0.5780	0.7093	0.6358	0.3481	0.3548	0.948
Extra Trees Classifier	0.6814	0.7168	0.5963	0.7089	0.6473	0.3610	0.3657	0.248
Linear Discriminant Analysis	0.6787	0.7061	0.5229	0.7510	0.6148	0.3541	0.3723	0.042
Decision Tree Classifier	0.6291	0.6166	0.5413	0.6459	0.5882	0.2559	0.2594	0.016
K Neighbors Classifier	0.5858	0.6072	0.2385	0.7465	0.3611	0.1622	0.2201	0.060
Quadratic Discriminant Analysis	0.5272	0.5329	0.8606	0.5750	0.6255	0.0662	0.0992	0.052
SVM - Linear Kernel	0.6444	0.0000	0.6257	0.7345	0.6213	0.2900	0.3242	0.040
Ridge Classifier	0.6931	0.0000	0.5450	0.7668	0.6349	0.3832	0.4013	0.012

Cohort 2: (5-fold CV Result)

Model	Accuracy	AUC	Recall	Prec.	F1	Kappa	MCC	TT (Sec)
CatBoost Classifier	0.6295	0.6699	0.5296	0.6644	0.5886	0.2596	0.2654	6.564
Gradient Boosting Classifier	0.6179	0.6683	0.4854	0.6638	0.5602	0.2366	0.2460	0.550
Logistic Regression	0.6315	0.6669	0.5417	0.6631	0.5962	0.2636	0.2682	6.582
Ada Boost Classifier	0.6204	0.6614	0.5226	0.6525	0.5797	0.2415	0.2467	0.312
Extreme Gradient Boosting	0.6204	0.6518	0.5176	0.6554	0.5779	0.2415	0.2474	1.558
Linear Discriminant Analysis	0.6204	0.6509	0.4955	0.6637	0.5673	0.2416	0.2499	0.134
Light Gradient Boosting Machine	0.6164	0.6443	0.5347	0.6417	0.5831	0.2333	0.2367	0.452
Naive Bayes	0.5810	0.6396	0.2281	0.7854	0.3536	0.1647	0.2340	0.050
Random Forest Classifier	0.6073	0.6366	0.5286	0.6313	0.5752	0.2151	0.2184	0.468
Extra Trees Classifier	0.6048	0.6212	0.5387	0.6238	0.5779	0.2100	0.2122	0.510
K Neighbors Classifier	0.5507	0.5640	0.3035	0.6045	0.4033	0.1035	0.1192	0.282
Decision Tree Classifier	0.5543	0.5310	0.4492	0.5735	0.5031	0.1094	0.1127	0.080
Quadratic Discriminant Analysis	0.5078	0.5065	0.8211	0.5455	0.5805	0.0129	0.0018	0.130
SVM - Linear Kernel	0.5876	0.0000	0.4492	0.7409	0.4866	0.1764	0.2310	0.098
Ridge Classifier	0.6285	0.0000	0.5116	0.6707	0.5803	0.2577	0.2654	0.044

Cohort 3: (5-fold CV Result)

Model	Accuracy	AUC	Recall	Prec.	F1	Kappa	MCC	TT (Sec)
Gradient Boosting Classifier	0.6035	0.6569	0.5157	0.6292	0.5659	0.2075	0.2116	0.432
CatBoost Classifier	0.6064	0.6541	0.5343	0.6296	0.5769	0.2133	0.2168	7.624
Random Forest Classifier	0.6157	0.6520	0.5912	0.6236	0.6065	0.2317	0.2323	0.352
Extra Trees Classifier	0.5994	0.6512	0.5982	0.6020	0.5992	0.1989	0.1995	0.408
Naive Bayes	0.5761	0.6420	0.3171	0.6633	0.4221	0.1541	0.1810	0.050
Ada Boost Classifier	0.5924	0.6294	0.5145	0.6134	0.5591	0.1853	0.1883	0.220
Logistic Regression	0.5901	0.6290	0.5238	0.6076	0.5617	0.1806	0.1830	6.366
Light Gradient Boosting Machine	0.5948	0.6288	0.5529	0.6052	0.5773	0.1898	0.1908	0.324
Extreme Gradient Boosting	0.5819	0.6252	0.5366	0.5921	0.5627	0.1641	0.1650	1.302
Linear Discriminant Analysis	0.5866	0.6060	0.5006	0.6082	0.5483	0.1737	0.1771	0.124
K Neighbors Classifier	0.5522	0.5800	0.2939	0.6114	0.3961	0.1063	0.1241	0.240
Quadratic Discriminant Analysis	0.5545	0.5760	0.6497	0.5946	0.5655	0.1089	0.1172	0.088
Decision Tree Classifier	0.5271	0.5245	0.5227	0.5286	0.5252	0.0542	0.0544	0.080
SVM - Linear Kernel	0.5656	0.0000	0.5804	0.6149	0.5429	0.1303	0.1498	0.090
Ridge Classifier	0.5895	0.0000	0.5075	0.6109	0.5534	0.1795	0.1828	0.040

Cohort 4: (5-fold CV Result)

Model	Accuracy	AUC	Recall	Prec.	F1	Kappa	MCC	TT (Sec)
Gradient Boosting Classifier	0.5717	0.6032	0.5520	0.5741	0.5618	0.1434	0.1440	0.482
Ada Boost Classifier	0.5668	0.6004	0.5267	0.5726	0.5475	0.1337	0.1346	0.188
CatBoost Classifier	0.5604	0.5992	0.5549	0.5614	0.5577	0.1209	0.1212	6.952
Logistic Regression	0.5721	0.5984	0.5423	0.5779	0.5590	0.1444	0.1449	6.468
Linear Discriminant Analysis	0.5629	0.5918	0.5286	0.5682	0.5474	0.1259	0.1263	0.124
Random Forest Classifier	0.5585	0.5845	0.6142	0.5536	0.5820	0.1167	0.1177	0.436
Naive Bayes	0.5429	0.5796	0.7805	0.5383	0.6286	0.0847	0.0950	0.048
Light Gradient Boosting Machine	0.5546	0.5787	0.5471	0.5565	0.5512	0.1092	0.1095	0.394
Extra Trees Classifier	0.5565	0.5784	0.6161	0.5515	0.5818	0.1128	0.1138	0.412
Extreme Gradient Boosting	0.5546	0.5739	0.5287	0.5583	0.5424	0.1094	0.1098	1.656
Decision Tree Classifier	0.5429	0.5361	0.5549	0.5434	0.5488	0.0857	0.0858	0.112
K Neighbors Classifier	0.5209	0.5333	0.4422	0.5262	0.4798	0.0424	0.0429	0.286
Quadratic Discriminant Analysis	0.5000	0.5002	0.4999	0.5117	0.4015	0.0004	0.0046	0.136
SVM - Linear Kernel	0.5317	0.0000	0.3484	0.5745	0.3405	0.0651	0.0783	0.112
Ridge Classifier	0.5692	0.0000	0.5316	0.5759	0.5524	0.1386	0.1391	0.114
