# Supplementary material for: A Multimorbidity Analysis of Hospitalized Patients With COVID-19 in Northwest Italy: Longitudinal Study Using Evolutionary Machine Learning and Health Administrative Data
Source: JMIR Public Health Surveill. 2024 Jul 18;10:e52353. doi: 10.2196/52353 (PMC11294776; doi:10.2196/52353)
Supplement: Multimedia Appendix 3 [file publichealth_v10i1e52353_app3.pdf]

Outcome Association of the Feature and the Support

Support: **The Occurrence of the Feature in the Evolutionarily Obtained Final Bin Dataset.**

Prevalence: **The Occurrence of the Feature in the Cohort Dataset.**

Cohort 1

Features	Chi-square	P value	Support	Prevalence
ATC R03BA	34.35	<.001	0.85	15.5
Age > 53	27.93	<.001	0.84	41.15
ATC N03AX	47.09	<.001	0.82	5.6
ATC R06AX	22.28	<.001	0.79	6.74
ATC J01XX	21.26	<.001	0.78	14.2
ATC C03CA	60.06	<.001	0.76	5.19
ATC N02AX	38.22	<.001	0.74	6.9
ATC A11CC	44.17	<.001	0.73	23.05
ATC C09CA	22.7	<.001	0.69	5.44
ICD 298	2	.16	0.68	0.16
ATC J01CA	14.37	<.001	0.66	14.12
ICD 411	1	.32	0.62	0.08
ATC J01EE	4.8	.03	0.61	2.44
ATC A02BX	27.17	<.001	0.57	7.47
ATC C08CA	32	<.001	0.57	5.84
ICD 550	2	.16	0.56	0.16
ATC B01AC	26.84	<.001	0.56	4.14
ATC D05AX	2.29	.13	0.55	2.27
ATC C07BB	1.92	.17	0.54	1.06
ATC A07EC	9	.003	0.54	2.03
ICD 618	1.8	.18	0.52	0.41
ICD 592	0.67	.41	0.51	0.49
ATC C07AA	17.19	<.001	0.5	1.7
ATC R06AE	17.31	<.001	0.5	4.22
ICD V54	2.67	.10	0.5	0.49
ATC B03BA	4	.05	0.5	1.3
ATC P01AB	23.06	<.001	0.49	2.76
ATC N03AE	11	<.001	0.48	0.89
ATC N06AB	12.58	<.001	0.48	13.07
ICD 278	7.36	.007	0.48	0.89
ICD 998	3	.08	0.47	0.24
ICD 574	8	.005	0.47	1.46
ATC B05BB	3	.08	0.47	0.24
ATC C09DA	17.82	<.001	0.46	3.57
ICD 038	8	.005	0.46	0.65
ICD V53	3	.08	0.46	0.24
ATC A03FA	8.91	.003	0.46	1.79
ATC C03DA	11	<.001	0.46	0.89
ICD 301	4	.05	0.44	0.32

ATC J01DC	2.29	.13	0.44	2.27
ATC J01AA	2.79	.09	0.44	2.35
ATC N04AA	0.2	.65	0.42	0.41
ATC N02AB	10	.002	0.42	0.81
ICD 482	3	.08	0.42	0.24
ICD 434	1	.32	0.42	0.08
ATC M05BA	0	1.0	0.42	0.65
ATC P01BA	3.6	.06	0.42	0.81
ICD 188	1	.32	0.4	0.08
ATC C09BB	15.21	<.001	0.4	1.54
ICD 218	0.06	.81	0.4	1.38
ICD 250	2	.16	0.4	0.16
ATC N05AD	1	.32	0.4	0.73
ICD 473	0.67	.41	0.4	0.49
ICD 571	1	.32	0.4	0.08
ATC N05AA	4	.05	0.38	0.32
ICD 812	2.67	.10	0.38	0.49
ICD 153	1	.32	0.38	0.08
ICD 553	1.8	.18	0.38	0.41
ICD 470	0.33	.56	0.38	0.24
ATC C01BD	3	.08	0.38	0.24
ATC A02BA	19.6	<.001	0.37	3.25
ATC A10AB	8	.005	0.37	0.65
ATC J05AB	0.08	.78	0.36	4.06
ATC S01ED	5.33	.02	0.36	0.97
ATC N02AJ	6	.01	0.36	0.49
ICD 780	4	.05	0.36	0.32
ATC N02BA	10.29	.001	0.36	1.14
ATC C03EA	29	<.001	0.36	2.35
ATC N03AF	3.6	.06	0.35	0.81
ATC L01BA	12	<.001	0.35	0.97
ATC C03BA	14.22	<.001	0.34	1.46
ICD 427	0.33	.56	0.34	0.24
ATC C10AB	5.44	.02	0.34	0.73
ATC C07AG	7	.008	0.34	0.57
ICD V71	1	.32	0.34	0.08
ATC N06AA	7.76	.005	0.34	2.35
ICD 296	9	.003	0.34	0.73
ICD 562	0	1.0	0.32	0.16
ICD 599	2	.16	0.32	0.16
ICD 717	0.09	.76	0.32	0.89
ICD 041	0	1.0	0.32	0.16
ATC C09BX	2.67	.10	0.32	0.49
ICD 560	1	.32	0.32	0.08
ICD 295	0.33	.56	0.31	0.24
ATC A12AX	20.55	<.001	0.3	4.3
ATC A10BB	9	.003	0.3	0.73

ATC C02AC	5	.03	0.3	0.41
ICD 428	1	.32	0.3	0.08
ICD 440	1	.32	0.3	0.08
ICD 735	0.08	.78	0.3	1.06
ICD 722	3	.08	0.3	0.24
ICD V56	5	.03	0.3	0.41
ATC M04AA	21	<.001	0.3	1.7
ICD V57	3	.08	0.3	0.24
ICD 726	0	1.0	0.29	0.65
ATC C10BA	9	.003	0.28	0.73
ATC R03AC	22.27	<.001	0.28	4.46
ATC N05AH	6	.01	0.28	0.49
ICD 454	1.47	.23	0.27	1.38
ATC R03AL	13.76	<.001	0.26	1.7
ICD 996	0.33	.56	0.26	0.24
ATC S01EC	0.14	.71	0.26	0.57
ICD 455	0	1.0	0.25	0.32
ATC C01DA	2	.16	0.24	0.16
ICD 820	2	.16	0.24	0.16
ICD 438	1	.32	0.24	0.08
ATC C03EB	0.2	.65	0.24	0.41
ICD 211	2	.16	0.24	0.16
ATC R03BB	8	.005	0.24	0.65
ICD 727	0.73	.39	0.24	1.79
ATC C09AA	34.2	<.001	0.24	7.71
ICD V43	1	.32	0.24	0.08
ATC N03AG	8.89	.003	0.23	1.54
ATC A12BA	13	<.001	0.23	1.06
ICD 162	1	.32	0.23	0.08
ICD V64	0.14	.71	0.23	0.57
ATC A07EA	2.27	.13	0.22	0.89
ICD 241	2	.16	0.22	0.16
ATC R03DA	5	.03	0.22	0.41
ATC N01BB	0.82	.37	0.22	0.89
ICD 786	1	.32	0.22	0.08
ATC A10BA	28.9	<.001	0.21	3.25
ATC B01AB	12.94	<.001	0.21	5.28
ICD 437	1	.32	0.2	0.08
ICD 995	0	1.0	0.2	0.16
ICD 366	1	.32	0.2	0.08
ICD 410	2	.16	0.2	0.16
ICD V58	20	<.001	0.2	1.62
ICD 585	5	.03	0.2	0.41
ICD 478	0.33	.56	0.2	0.24
ICD 626	0	1.0	0.2	0.49
ATC G03DB	0.1	.75	0.2	3.25
ATC C01BC	0.33	.56	0.19	0.24

ICD 518	6	.01	0.19	0.49
ATC C02CA	12.25	<.001	0.18	1.3
ATC B01AA	8	.005	0.18	0.65
ATC G03CA	1.88	.17	0.18	2.76
ICD 174	1.92	.17	0.17	1.06
ICD 354	2	.16	0.17	0.16
ATC N06AX	55.85	<.001	0.16	6.33
ATC N02CC	4.45	.03	0.16	5.28
ATC C10AX	4.45	.03	0.15	0.89
ATC S01EE	1	.32	0.14	0.32
ICD 424	3	.08	0.14	0.24
ATC B03BB	15.21	<.001	0.14	6.17
ATC C03AA	2.67	.10	0.14	0.49
ATC A05AA	5.26	.02	0.13	1.87
ATC C07AB	31.27	<.001	0.13	9.66
ATC B03AA	2.25	.13	0.13	11.69
ATC B02AA	4.45	.03	0.12	3.57
ATC A12AA	15	<.001	0.12	1.22
ATC C09BA	14.4	<.001	0.12	3.25
ATC G03AA	4.8	.03	0.12	2.44
ICD 338	4.5	.03	0.12	0.65
ICD 728	1	.32	0.12	0.08
ATC M01AH	23.43	<.001	0.11	9.01
ATC J02AC	16.03	<.001	0.1	10.71
ATC J01MA	27.1	<.001	0.1	27.03
ATC M01AB	26.85	<.001	0.1	23.94
ICD 486	2	.16	0.09	0.16
ATC N02AA	30.6	<.001	0.09	6.9
ATC H03AA	19.56	<.001	0.09	10.8
ATC A07AA	48.13	<.001	0.08	9.74
ATC C10AA	33.2	<.001	0.08	4.95
ICD 621	2.45	.12	0.08	2.68
ATC N02BE	22.23	<.001	0.07	9.5
ATC M01AX	6.75	.009	0.06	3.9
ATC J01FA	17.93	<.001	0.06	35.06
ATC H02AB	26.89	<.001	0.06	41.31
ATC R03AK	13.04	<.001	0.05	8.52
ATC J01CR	24.55	<.001	0.04	44.48

Cohort 2

Features	Chi-square	P value	Support	Prevalence
ATC A10BA	39.17	<.001	0.86	4.31
ATC N02BE	21.45	<.001	0.79	6.4
ATC J05AB	14.06	<.001	0.76	2.91
ATC C03CA	48.4	<.001	0.76	4.09
ATC M04AA	73.28	<.001	0.74	5.13
ATC C09CA	18.82	<.001	0.71	8.4
ATC C02CA	33.82	<.001	0.65	3.22
ATC C08CA	42.26	<.001	0.65	7.4
ICD V64	0	1.0	0.64	0.18
ICD V54	1.29	.26	0.64	0.32
ATC J02AC	4.97	.03	0.64	6.18
ICD 188	1	.32	0.63	0.18
ATC N06AB	4.45	.03	0.63	8.58
ATC S01EE	3.27	.07	0.62	0.68
ATC N03AG	3.07	.08	0.61	2.5
ATC M01AB	10.54	.001	0.6	18.21
ICD 735	0	1.0	0.6	0.18
ICD 454	0.04	.83	0.6	1.04
ATC N03AE	1.88	.17	0.6	1.54
ICD 820	1	.32	0.6	0.05
ATC B01AA	6.37	.01	0.59	0.86
ICD 211	0.2	.65	0.56	0.23
ATC P01AB	11.76	<.001	0.56	1.54
ICD 574	0.2	.65	0.56	0.91
ATC A07EC	8.64	.003	0.56	2.54
ATC C09BX	3.86	.05	0.56	0.95
ICD 482	8	.005	0.55	0.36
ATC B03AA	15.75	<.001	0.55	2.77
ICD V56	6	.01	0.55	0.27
ATC M01AC	6	.01	0.55	2.45
ICD 550	0	1.0	0.55	2.27
ATC G04CB	11.64	<.001	0.54	1
ICD 427	0.14	.71	0.54	0.32
ICD 571	2.67	.10	0.53	0.27
ICD V53	1.33	.25	0.52	0.54
ICD 428	7	.008	0.52	0.32
ICD 813	3	.08	0.52	0.14
ICD 276	1	.32	0.51	0.05
ATC S01ED	7.76	.005	0.51	1.32
ATC N02AX	8.85	.003	0.51	4.31
ATC R03AL	12.46	<.001	0.5	1.18

ATC N05AH	0.62	.43	0.5	1.18
ICD 278	0	1.0	0.5	0.45
ICD 996	5.44	.02	0.5	0.41
ICD 440	0.33	.56	0.5	0.14
ICD 727	1.64	.2	0.5	1
ICD 410	14	<.001	0.48	0.64
ATC N02CC	0.02	.88	0.48	1.86
ATC C07AG	7.12	.008	0.48	0.77
ICD 995	0.67	.41	0.48	0.27
ICD 518	6.23	.01	0.47	0.59
ATC J01EE	24.07	<.001	0.47	2.72
ATC B02AA	4.76	.03	0.46	0.77
ATC C07BB	0.6	.44	0.46	0.68
ICD 812	1	.32	0.46	0.18
ICD V57	0.33	.56	0.46	0.14
ICD 038	5.33	.02	0.45	0.54
ICD 600	0.09	.76	0.45	0.5
ATC C03AA	4.5	.03	0.45	0.36
ATC N04AA	1.09	.3	0.44	1.04
ATC B03BB	7.18	.007	0.44	3.95
ATC C01DA	18	<.001	0.44	0.82
ATC C03BA	0.06	.81	0.44	0.77
ATC C01BC	0.14	.71	0.44	0.32
ICD 478	0.2	.65	0.43	0.23
ATC C09BB	1.47	.23	0.43	3.09
ATC M05BA	2	.16	0.43	0.09
ICD V43	0	1.0	0.43	0.36
ICD 470	4.45	.03	0.42	0.5
ATC A12AX	0.39	.53	0.42	1.04
ICD 250	1	.32	0.42	0.18
ICD 715	0.89	.35	0.42	0.82
ICD 415	0.33	.56	0.42	0.14
ICD 780	0.2	.65	0.42	0.23
ATC L01BA	0.4	.53	0.42	0.45
ATC J01DC	0.17	.68	0.42	1.09
ICD 726	0.14	.71	0.41	0.32
ICD 414	6	.01	0.41	0.27
ATC P01BA	3	.08	0.4	0.14
ATC G03DB	1	.32	0.4	0.05
ATC C03DA	24.14	<.001	0.4	1.27
ATC H02AB	24.33	<.001	0.4	33.51
ICD 585	11	<.001	0.4	0.5
ATC C03EB	8.33	.004	0.4	0.54
ATC A05AA	0	1.0	0.4	1.27
ATC N03AA	2.57	.11	0.4	0.64
ATC N02BA	25.97	<.001	0.4	1.68
ICD 162	1	.32	0.39	0.05

ICD 296	1.8	.18	0.39	0.23
ICD 584	0.33	.56	0.39	0.14
ICD 413	12	<.001	0.39	0.54
ATC C02AC	9.31	.002	0.39	0.59
ATC N02AJ	4.45	.03	0.38	0.5
ATC R03BB	15.21	<.001	0.38	0.86
ATC C09DB	0.95	.33	0.38	1.73
ATC H03AA	2.2	.14	0.38	2.5
ICD V71	0.2	.65	0.38	0.23
ICD 366	0.33	.56	0.37	0.14
ICD 295	1	.32	0.37	0.41
ATC A10BB	11.57	<.001	0.37	1.27
ICD V58	15.7	<.001	0.37	1.04
ATC C10AB	3.27	.07	0.36	2
ICD 599	2	.16	0.35	0.09
ICD 998	1.8	.18	0.35	0.23
ATC N05AA	0.2	.65	0.34	0.91
ATC R03DC	9.94	.002	0.34	0.77
ATC C10BA	0.6	.44	0.34	0.68
ICD 354	0.33	.56	0.34	0.14
ATC S01EC	3.6	.06	0.33	0.45
ATC J01XX	20.32	<.001	0.33	4.13
ICD 553	18	<.001	0.33	0.82
ICD 331	1	.32	0.32	0.05
ATC A12AA	16.2	<.001	0.32	0.91
ICD 786	2.67	.10	0.32	0.27
ICD 486	2.67	.10	0.32	0.27
ATC N01BB	2.27	.13	0.32	0.5
ATC N02AB	6.4	.01	0.32	0.45
ICD 241	0.2	.65	0.31	0.23
ICD 338	2.67	.10	0.3	0.27
ATC C09AA	25.47	<.001	0.3	11.99
ICD 437	3	.08	0.3	0.14
ICD 434	6	.01	0.3	0.27
ICD 153	2	.16	0.3	0.09
ATC M01AX	3.56	.06	0.3	3.27
ATC N05AD	1.38	.24	0.3	1.18
ATC R03DA	0.82	.37	0.29	0.5
ICD 455	0.33	.56	0.29	0.54
ICD 301	0.14	.71	0.29	0.32
ICD 041	0.33	.56	0.29	0.14
ICD 431	2.67	.10	0.29	0.27
ATC D05AX	0.02	.89	0.29	2.41
ATC A02AD	13.26	<.001	0.28	6.63
ICD 411	3	.08	0.28	0.14
ATC C07AA	3.6	.06	0.28	0.45
ATC B05BB	0.09	.76	0.28	0.5

ATC N06AA	1.09	.3	0.26	1.04
ATC A10AB	16.03	<.001	0.26	1.77
ATC A02BA	3.46	.06	0.26	1.59
ATC C09DA	17.31	<.001	0.26	5.31
ICD 717	0.29	.59	0.26	1.41
ATC A02BX	8.89	.003	0.25	5.9
ATC C09BA	0.76	.38	0.25	3.81
ATC B01AB	20.18	<.001	0.24	5.4
ICD 424	4	.05	0.24	0.18
ICD 562	4	.05	0.24	0.18
ICD 722	0.5	.48	0.24	0.36
ATC A12BA	8.89	.003	0.24	0.86
ATC C10AX	8.64	.003	0.24	2.54
ATC N02AA	4.75	.03	0.24	4.63
ATC N06AX	0.04	.83	0.22	4.09
ICD 298	0.4	.53	0.22	0.45
ATC C03EA	6.25	.01	0.22	0.73
ICD 592	0.05	.83	0.22	0.95
ICD 473	0.33	.56	0.21	0.54
ICD 173	1.8	.18	0.21	0.23
ATC C01BD	4	.05	0.2	0.18
ATC A07EA	0.02	.88	0.2	1.86
ICD 728	0.67	.41	0.2	0.27
ICD 438	1.8	.18	0.2	0.23
ICD 185	0	1.0	0.2	0.09
ATC A07AA	27.58	<.001	0.2	9.76
ATC J01AA	0.93	.34	0.19	1.23
ATC N03AF	0.15	.69	0.18	1.18
ATC R03AC	8.01	.005	0.16	4.13
ATC A03FA	1.64	.2	0.14	1
ATC J01FA	6.48	.01	0.14	28.7
ATC J01CA	7.31	.007	0.12	11.49
ATC R03BA	8.74	.003	0.11	12.99
ATC R06AE	0.41	.52	0.11	2.77
Age > 53	30.77	<.001	0.1	44.69
ATC A11CC	24.66	<.001	0.1	6.63
ATC R06AX	5.84	.02	0.1	4.86
ATC J01CR	10.38	.001	0.1	40.33
ATC M01AH	10.78	.001	0.09	4.59
ATC M01AE	16.79	<.001	0.09	15.62
ATC N03AX	28.51	<.001	0.07	5
ATC R03AK	12.45	<.001	0.06	7.72
ATC J01DD	18.8	<.001	0.06	15.85
ATC G04CA	25.94	<.001	0.06	6.95

Cohort 3

Features	Chi-square	P value	Support	Prevalence
ATC N02AX	26.56	<.001	0.84	12.96
ATC M04AA	74.69	<.001	0.82	8.5
ATC C03EA	15.69	<.001	0.76	5.35
ATC A02BA	8.12	.004	0.75	4.04
ATC B01AB	16.02	<.001	0.73	12.59
ATC N03AX	26.59	<.001	0.7	11.7
ICD 295	4.57	.03	0.68	0.73
ICD 813	0.25	.62	0.68	0.84
ATC N02AA	26	<.001	0.68	13.9
ATC J05AB	7.13	.008	0.65	5.77
ATC A12AA	2.53	.11	0.62	4.67
ATC C07BB	2	.16	0.62	2.62
ATC B03BB	17.82	<.001	0.61	9.23
ATC R03AC	7.95	.005	0.6	7.19
ICD 427	4.55	.03	0.58	1.15
ICD 413	0.14	.71	0.56	0.37
ICD V53	9.31	.002	0.56	0.68
ATC A12BA	7.05	.008	0.56	3.93
ICD 518	9.8	.002	0.56	2.36
ICD 574	1.4	.24	0.55	1.84
ATC J01DC	3.31	.07	0.55	2.68
ATC N06AA	2.19	.14	0.55	4.04
ATC A05AA	7.12	.008	0.54	3.57
ATC G03AA	1	.32	0.53	0.05
ATC C01DA	14.88	<.001	0.52	2.57
ICD 618	0	1.0	0.5	1.15
ICD 727	0.11	.74	0.5	1.89
ICD 434	0.14	.71	0.5	0.37
ICD 553	0.2	.65	0.5	0.26
ATC G04CA	0	1.0	0.49	0.1
ICD 354	1	.32	0.48	0.05
ICD 241	3.6	.06	0.48	0.52
ICD 038	15.11	<.001	0.48	1.84
ICD 455	0.33	.56	0.47	0.16
ATC N02AJ	8	.005	0.47	2.62
ATC R06AX	2.14	.14	0.47	5.51
ICD 478	0.14	.71	0.46	0.37
ICD 599	0.14	.71	0.46	0.37
ICD 433	0.67	.41	0.46	0.31
ATC M01AE	6.76	.009	0.46	31.79
ICD 728	2.78	.10	0.44	0.47
ICD 717	0.69	.41	0.44	0.68
ICD 437	2.27	.13	0.43	0.58

ATC D05AX	1	.32	0.42	3.36
ATC R03BB	31.19	<.001	0.42	5.09
ICD 188	0.67	.41	0.42	0.31
ATC B05BB	0.12	.72	0.42	1.68
ICD 482	1.67	.2	0.42	0.79
ICD 331	0.07	.8	0.42	0.79
ATC B02AA	1.69	.19	0.41	1.52
ATC R03DA	7.26	.007	0.41	1.63
ATC A07EC	8.38	.004	0.4	4.56
ATC C09DA	14.42	<.001	0.4	14.9
ATC N02BE	17.1	<.001	0.4	17.26
ATC N06AB	9.34	.002	0.39	22.3
ICD 296	0.04	.84	0.39	1.31
ATC N03AA	0	1.0	0.39	1.57
ICD 153	0.5	.48	0.39	0.42
ICD 550	0.11	.74	0.39	0.47
ICD 438	0	1.0	0.39	0.42
ICD 470	1	.32	0.39	0.21
ICD 428	5.26	.02	0.39	1.21
ICD 214	0.2	.65	0.38	0.26
ATC B03BA	15.08	<.001	0.38	2.73
ATC C07AG	17.29	<.001	0.38	1.47
ATC J01AA	4.5	.03	0.38	1.68
ATC C01BC	0.36	.55	0.38	1.31
ICD V43	2.19	.14	0.37	1.94
ATC N03AF	1.29	.26	0.37	1.47
ATC S01EC	0.26	.61	0.37	1.84
ATC M01AC	1.06	.3	0.37	7.14
ATC N05AH	0.03	.86	0.36	1.63
ICD 812	1.33	.25	0.36	0.63
ATC P01AB	1.14	.29	0.36	2.26
ICD 780	2.67	.10	0.36	0.31
ICD 298	1.8	.18	0.36	0.26
ICD 486	4.57	.03	0.35	0.73
ICD V71	2.78	.10	0.35	0.47
ATC R03DC	3.24	.07	0.34	1.31
ATC C07AA	0	1.0	0.34	2.2
ICD 626	0.33	.56	0.34	0.16
ATC N03AE	0.38	.54	0.34	2.2
ICD 820	3.52	.06	0.33	1.21
ICD V57	0.11	.74	0.32	0.47
ICD 424	2.27	.13	0.32	0.58
ICD 174	0.67	.41	0.32	1.26
ATC A12AX	5.93	.01	0.32	12.12
ICD V54	2	.16	0.32	0.42
ICD 338	2.29	.13	0.32	1.47
ICD 366	1.67	.2	0.32	0.79

ATC G03CA	0.09	.77	0.32	5.3
ICD 621	1.29	.26	0.3	1.47
ATC N02AB	4.79	.03	0.3	2.47
ATC C01BD	1.32	.25	0.3	1
ICD 571	3.57	.06	0.3	0.37
ICD 211	4.5	.03	0.3	0.42
ICD 414	3.6	.06	0.29	0.52
ATC A11CC	2.45	.12	0.29	45.33
ATC C09AA	2.58	.11	0.28	19.57
ATC M01AH	3.92	.05	0.28	18.31
ICD 410	13.5	<.001	0.28	1.26
ICD 998	1.8	.18	0.28	0.26
ATC G03DB	1	.32	0.28	0.05
ATC S01ED	0.05	.82	0.27	3.99
ATC J02AC	9.88	.002	0.27	8.5
ICD V58	7.37	.007	0.26	2.57
ATC N01BB	5.54	.02	0.26	1.36
ICD 560	2	.16	0.26	0.42
ATC N03AG	0	1.0	0.26	3.46
ATC C10AB	2.31	.13	0.26	1.84
ATC S01EE	2.94	.09	0.26	1.78
ATC C03DA	28.41	<.001	0.26	4.62
ICD 995	4.45	.03	0.26	0.58
ATC C10BA	9.38	.002	0.25	2.47
ICD V56	2.27	.13	0.25	0.58
ATC M05BA	3.18	.07	0.24	5.35
ICD 278	2.91	.09	0.24	1.15
ICD 250	4.45	.03	0.24	0.58
ICD 218	2.67	.10	0.24	0.31
ATC J01EE	11.23	<.001	0.24	5.09
ICD 431	0	1.0	0.23	0.21
ATC A07EA	5	.03	0.23	1.05
ATC N06AX	7.74	.005	0.23	13.12
ATC N02CC	1.92	.17	0.22	2.73
ATC C09DB	2.81	.09	0.22	2.26
ATC C03BA	1.92	.17	0.22	3.31
ICD 996	2.67	.10	0.21	1.26
ICD 162	0	1.0	0.21	0.21
ICD 041	1.8	.18	0.2	0.26
ATC C02AC	16.67	<.001	0.2	1.26
ICD 786	0	1.0	0.2	0.42
ATC M01AB	2.66	.10	0.2	33.11
ICD 584	4.5	.03	0.2	0.42
ICD 440	3	.08	0.19	0.16
ICD 473	1	.32	0.19	0.21
ATC H03AA	1.27	.26	0.19	16.58
ICD 735	0.36	.55	0.18	1.31

ICD 415	0.4	.53	0.18	0.52
ATC C03AA	6.12	.01	0.18	1.68
ICD 411	6.4	.01	0.18	0.52
ATC R03BA	3.56	.06	0.18	22.4
ATC C02CA	24.31	<.001	0.18	5.61
ATC C09BB	1.42	.23	0.17	4.46
ICD 592	0.4	.53	0.16	0.52
ATC C08CA	28.69	<.001	0.16	16.16
ICD V64	2	.16	0.16	0.42
ICD 715	1.92	.17	0.16	3.31
ATC C09BX	2.91	.09	0.16	1.15
ATC J01CR	6.04	.01	0.16	54.2
ATC N04AA	0.03	.86	0.16	1.63
ATC C03EB	36	<.001	0.16	3.36
ATC R06AE	6.45	.01	0.16	5.93
ATC M01AX	4.17	.04	0.16	8.5
ATC N05AD	0.45	.5	0.16	2.89
ICD 722	1.8	.18	0.15	0.26
ATC J01DD	5.06	.02	0.14	29.12
ATC C09BA	2	.16	0.14	8.5
ATC C09CA	7.84	.005	0.14	15.42
ATC B01AC	27.49	<.001	0.14	21.04
ICD 562	0.09	.76	0.14	0.58
ICD 726	0.11	.74	0.14	0.47
ATC L01BA	2.31	.13	0.14	1.84
ICD 173	2	.16	0.13	0.42
ATC A03FA	1.03	.31	0.12	3.25
ATC N05AA	0.03	.86	0.12	1.63
ATC A10BB	18	<.001	0.12	3.78
ATC B01AA	16.25	<.001	0.12	3.52
ATC J01CA	9	.003	0.12	17
ATC C10AX	10.32	.001	0.1	3.99
ATC A02AD	2.93	.09	0.1	15.06
ATC A02BC	29.31	<.001	0.1	49.32
ATC A10AB	20.75	<.001	0.1	4.25
ATC R03AL	4.09	.04	0.1	2.89
ICD 276	1.29	.26	0.09	0.37
ATC C07AB	10.98	<.001	0.09	24.76
ATC C03CA	49.26	<.001	0.08	19.99
ATC A02BX	8.84	.003	0.08	12.01
ATC J01FA	5.1	.02	0.08	38.25
ICD 454	0	1.0	0.08	1.15
ATC A10BA	21.94	<.001	0.07	11.39
ATC R03AK	25.5	<.001	0.07	15.22
ATC A07AA	30.27	<.001	0.06	17.68
Age > 68	28.96	<.001	0.06	38.61
ATC B03AA	9.57	.002	0.06	7.5

ATC J01XX	9.99	.002	0.06	21.51
ATC N02BA	7.44	.006	0.06	5.93
ATC C10AA	16.53	<.001	0.05	26.29
ATC J01MA	11.02	<.001	0.04	41.19
ATC H02AB	9.95	.002	0.03	47.59

Cohort 4

Features	Chi-square	P value	Support	Prevalence
ATC G04CA	5.53	.02	0.8	25.75
ATC J01CA	6.98	.008	0.73	14.47
ATC C09DA	3.21	.07	0.66	13.11
ATC C09AA	4.51	.03	0.66	26.32
ATC B01AA	10.37	.001	0.64	4.61
ATC C03CA	9.57	.002	0.62	16.49
ICD 995	0	1.0	0.61	0.44
ATC N04AA	15.7	<.001	0.59	1.01
ICD 153	2.25	.13	0.57	0.7
ATC J02AC	5.44	.02	0.57	6.32
ICD 437	0.4	.53	0.56	0.44
ATC C09BX	1.47	.22	0.56	2.41
ICD 250	0	1.0	0.55	0.53
ICD 298	2	.16	0.55	0.35
ATC N05AH	8.17	.004	0.55	1.05
ATC A10BA	21.77	<.001	0.54	16.32
ICD 415	1	.32	0.52	0.39
ATC C07AG	5.45	.02	0.52	2.32
ICD 786	0.69	.41	0.5	0.57
ICD 424	1.92	.17	0.5	0.57
ATC J05AB	7.22	.007	0.5	4.43
ATC C03EB	0.53	.47	0.5	2.06
ICD 780	1.32	.25	0.5	0.83
ICD 592	3	.08	0.5	0.53
ICD 185	0.05	.82	0.49	0.83
ICD 722	2.25	.13	0.48	0.7
ATC C03DA	10.45	.001	0.48	5.75
ATC H03AA	0.36	.55	0.48	4.39
ICD 428	2.78	.10	0.48	1.58
ICD 473	0.69	.41	0.47	0.57
ATC N05AA	17.06	<.001	0.47	1.36
ATC A03FA	0	1.0	0.47	1.75
ICD 518	0.07	.79	0.46	2.54
ATC S01EE	1.61	.2	0.46	2.72
ICD 486	0	1.0	0.46	0.88
ICD 366	2.57	.11	0.46	0.61

ATC S01EC	0.1	.75	0.45	1.75
ATC A05AA	1.98	.16	0.45	2.68
ICD 585	0.05	.82	0.45	0.83
ATC C09CA	6.76	.009	0.45	17.54
ICD V71	8.33	.004	0.45	0.53
ATC D05AX	4.57	.03	0.44	4.65
ATC M01AX	2.04	.15	0.44	7.76
ATC B02AA	1.14	.29	0.44	2.46
ATC R03DC	0.25	.62	0.44	0.7
ATC A12AA	5.16	.02	0.43	1.67
ICD 413	4.25	.04	0.42	2.32
ATC G04CB	1.82	.18	0.42	9.65
ATC L01BA	7.76	.005	0.42	1.27
ICD 454	0.07	.8	0.42	0.66
ICD 296	1.47	.23	0.41	0.75
ICD 162	2.27	.13	0.41	0.48
ATC C03BA	2.48	.12	0.41	2.54
ICD 574	0.02	.88	0.41	1.89
ATC N02AA	7.69	.006	0.4	9.12
ATC A07EC	1.22	.27	0.4	3.6
ICD 715	0.8	.37	0.4	2.68
ICD 726	0.5	.48	0.4	0.35
ICD 295	2.58	.11	0.4	0.83
ATC J01EE	2.6	.11	0.4	4.87
ATC P01BA	4	.05	0.4	0.7
ICD 431	0.25	.62	0.4	0.7
ATC C02CA	0.56	.45	0.4	7.81
ATC R03AK	2.1	.15	0.4	13.03
ICD 188	0.03	.86	0.4	1.36
ICD 038	2.5	.11	0.39	1.75
ICD 550	0.71	.4	0.38	3.95
ICD 433	1.8	.18	0.38	0.88
ICD 470	1.8	.18	0.38	0.22
ICD 584	2.57	.11	0.38	0.61
ICD 411	1.32	.25	0.38	1.62
ICD 478	2	.16	0.38	0.35
ICD 455	0.08	.78	0.38	0.57
ATC N05AD	3.92	.05	0.37	2.19
ICD V56	3.86	.05	0.37	0.92
ICD 571	7.12	.008	0.36	0.75
ATC S01ED	0.46	.5	0.36	4.69
ATC A02BA	0.6	.44	0.36	3.55
ATC C07BB	0.02	.88	0.36	1.8
ICD 440	0.13	.72	0.36	1.32
ATC R03DA	0.57	.45	0.36	1.23
ICD V43	2.61	.11	0.36	1.36
ATC C10AB	8.73	.003	0.36	2.89

ATC J01AA	1.52	.22	0.36	1.84
ICD 560	0.4	.53	0.35	0.44
ICD 482	0.18	.67	0.35	0.96
ICD 813	1	.32	0.34	0.18
ICD 735	0.2	.65	0.34	0.22
ICD 041	1	.32	0.34	0.39
ICD 812	0.14	.71	0.34	0.31
ATC C01BC	1.59	.21	0.34	2.24
ICD 728	0.08	.78	0.34	0.57
ATC C03AA	2.13	.14	0.34	1.01
ATC N06AB	1.9	.17	0.34	12.24
ICD V64	0.07	.8	0.33	0.66
ATC A12AX	0.07	.8	0.32	2.63
ATC C01DA	14.72	<.001	0.32	4.08
ATC N02CC	0.15	.69	0.32	1.14
ATC N03AA	17.64	<.001	0.32	1.1
ICD 996	1.69	.19	0.32	1.27
ICD V53	0	1.0	0.32	0.88
ICD 338	0.22	.64	0.32	0.79
ATC C10BA	0.6	.44	0.31	3.55
ICD 174	1	.32	0.31	0.04
ICD 427	0.02	.88	0.31	1.89
ICD 301	1	.32	0.3	0.18
ATC A02BX	1.83	.18	0.3	8.64
ICD 331	9.94	.002	0.3	0.75
ICD 727	1.14	.29	0.3	1.89
ATC J01FA	2.1	.15	0.29	33.42
ATC C03EA	1	.32	0.28	2.81
ATC A10AB	3.04	.08	0.28	6.36
ATC N03AE	0.49	.48	0.28	2.24
ICD 998	0.14	.71	0.28	0.31
ICD 562	0.4	.53	0.28	0.44
ATC C09DB	0.89	.35	0.28	3.16
ATC R03BB	1.75	.19	0.28	4.91
ICD 553	0.25	.62	0.28	0.7
ICD 414	4.79	.03	0.28	2.06
ATC B03BB	0.02	.89	0.28	8.86
ATC B01AB	0.84	.36	0.28	11.8
ICD 599	1.14	.29	0.28	0.61
ATC A07AA	2.14	.14	0.28	16.05
ATC N03AX	2.96	.09	0.27	10
ATC A10BB	20.18	<.001	0.27	5.88
ICD 214	0	1.0	0.27	0.35
ATC A07EA	0.76	.38	0.26	3.68
ICD 820	2.58	.11	0.26	0.83
ICD 211	0	1.0	0.26	0.35
ATC N02BA	0.67	.41	0.26	9.39

ATC R03AL	2.8	.09	0.26	3.07
ICD V54	4	.05	0.26	0.18
ATC N03AG	7.78	.005	0.26	3.25
ATC N02AB	0.47	.49	0.24	1.49
ICD 717	0.2	.65	0.24	0.88
ICD V57	0	1.0	0.24	0.88
ATC C07AA	0.16	.69	0.24	2.41
ATC R06AX	0.17	.68	0.24	4.12
ATC N01BB	0.6	.44	0.24	0.66
ATC N02AX	3.36	.07	0.24	9.52
ATC M05BA	1	.32	0.24	0.39
ATC P01AB	0.09	.76	0.24	1.93
ICD 434	0.4	.53	0.22	1.75
ICD 241	2	.16	0.22	0.09
ATC N02BE	8.4	.004	0.22	11.05
ICD 438	1.81	.18	0.22	1.18
ATC M01AC	5.54	.02	0.22	4.56
ATC N03AF	2.94	.09	0.22	1.49
ATC N06AA	0	1.0	0.21	2.19
ATC J01DC	2.69	.10	0.21	1.97
ICD V58	0.31	.58	0.2	2.28
ATC C01BD	9	.003	0.2	2.15
ATC B03BA	0.78	.38	0.2	2.76
ICD 173	0.09	.76	0.2	0.48
ATC N06AX	6.68	.01	0.19	8.51
ATC B05BB	1.69	.19	0.19	1.27
ICD 278	0	1.0	0.18	0.35
ATC A02AD	2.07	.15	0.18	9.34
ATC C07AB	3.03	.08	0.18	27.98
ICD 410	4.13	.04	0.18	2.72
ATC A12BA	0.75	.39	0.17	2.85
ATC R03AC	2.67	.10	0.16	5.92
ATC B03AA	0.23	.63	0.16	6.93
ICD 600	0.4	.53	0.16	2.76
ATC R06AE	0.06	.81	0.16	3.16
ICD 354	0.14	.71	0.15	0.31
ATC J01XX	0.95	.33	0.14	6.67
ATC C09BB	4.06	.04	0.14	6.23
ATC B01AC	13.78	<.001	0.14	31.18
ATC C10AX	12.91	<.001	0.12	6.58
ATC C08CA	8.5	.004	0.12	21.14
ATC M01AH	0.8	.37	0.12	10.79
ATC J01DD	8.15	.004	0.12	24.17
ICD 276	0.07	.8	0.12	0.66
ATC M01AB	2.23	.14	0.12	26.97
ATC J01MA	3.39	.07	0.12	40.53
ATC N02AJ	0.13	.72	0.1	1.32

ATC J01CR	1.83	.18	0.09	48.46
ATC C10AA	18.2	<.001	0.09	31.32
ATC C09BA	5.12	.02	0.08	9.91
ATC H02AB	6.69	.01	0.08	36.89
ATC M04AA	22.32	<.001	0.08	16.27
ATC M01AE	10.7	.001	0.06	25.57
ATC R03BA	8.68	.003	0.06	17.59
ATC A02BC	11.14	<.001	0.05	49.39
Age > 68	16.64	<.001	0.04	40.53
ATC A11CC	4.55	.03	0.03	13.2
