# Supplementary material for: A Multimorbidity Analysis of Hospitalized Patients With COVID-19 in Northwest Italy: Longitudinal Study Using Evolutionary Machine Learning and Health Administrative Data
Source: JMIR Public Health Surveill. 2024 Jul 18;10:e52353. doi: 10.2196/52353 (PMC11294776; doi:10.2196/52353)
Supplement: Multimedia Appendix 4 [file publichealth_v10i1e52353_app4.pdf]

Most Prevalent Multimorbidity Feature Combinations in Evolved Bins

Cohort 1

Support (s_{min}=0.5)	Length	Frequent Item sets
0.85	1	{'code_drug_R03BA'}
0.84	1	{'eta_53above'}
0.82	1	{'code_drug_N03AX'}
0.79	1	{'code_drug_R06AX'}
0.78	1	{'code_drug_J01XX'}
0.76	1	{'code_drug_C03CA'}
0.74	1	{'code_drug_N02AX'}
0.73	1	{'code_drug_A11CC'}
0.69	1	{'code_drug_C09CA'}
0.68	1	{'code_diag_298'}
0.66	1	{'code_drug_J01CA'}
0.62	1	{'code_diag_411'}
0.61	1	{'code_drug_J01EE'}
0.57	1	{'code_drug_C08CA'}
0.57	1	{'code_drug_A02BX'}
0.56	1	{'code_drug_B01AC'}
0.56	1	{'code_diag_550'}
0.55	1	{'code_drug_D05AX'}
0.54	1	{'code_drug_C07BB'}
0.54	1	{'code_drug_A07EC'}
0.52	1	{'code_diag_618'}
0.51	1	{'code_diag_592'}
0.5	1	{'code_drug_C07AA'}
0.5	1	{'code_drug_R06AE'}
0.74	2	{'eta_53above', 'code_drug_R03BA'}
0.72	2	{'eta_53above', 'code_drug_N03AX'}
0.72	2	{'code_drug_N03AX', 'code_drug_R03BA'}
0.68	2	{'code_drug_R06AX', 'code_drug_R03BA'}
0.68	2	{'code_drug_R06AX', 'eta_53above'}
0.68	2	{'code_drug_R03BA', 'code_drug_J01XX'}
0.67	2	{'code_drug_R06AX', 'code_drug_N03AX'}
0.65	2	{'code_drug_N03AX', 'code_drug_J01XX'}
0.65	2	{'eta_53above', 'code_drug_J01XX'}
0.64	2	{'eta_53above', 'code_drug_N02AX'}
0.64	2	{'code_drug_C03CA', 'eta_53above'}
0.64	2	{'code_drug_R03BA', 'code_drug_N02AX'}
0.64	2	{'code_drug_C03CA', 'code_drug_R03BA'}
0.63	2	{'code_drug_C03CA', 'code_drug_N03AX'}
0.63	2	{'code_drug_R06AX', 'code_drug_J01XX'}
0.62	2	{'code_drug_A11CC', 'code_drug_R03BA'}
0.62	2	{'eta_53above', 'code_drug_A11CC'}
0.61	2	{'code_drug_R06AX', 'code_drug_C03CA'}

0.61	2	{'code_drug_N03AX', 'code_drug_N02AX'}
0.61	2	{'code_drug_R06AX', 'code_drug_N02AX'}
0.61	2	{'code_drug_A11CC', 'code_drug_N03AX'}
0.59	2	{'code_drug_C09CA', 'code_drug_R03BA'}
0.59	2	{'code_drug_C03CA', 'code_drug_J01XX'}
0.58	2	{'code_drug_A11CC', 'code_drug_J01XX'}
0.58	2	{'code_drug_C09CA', 'eta_53above'}
0.57	2	{'code_drug_C03CA', 'code_drug_N02AX'}
0.57	2	{'code_drug_R03BA', 'code_diag_298'}
0.57	2	{'code_drug_N03AX', 'code_diag_298'}
0.56	2	{'code_drug_R06AX', 'code_drug_A11CC'}
0.56	2	{'code_drug_R03BA', 'code_drug_J01CA'}
0.56	2	{'code_drug_C03CA', 'code_drug_A11CC'}
0.56	2	{'eta_53above', 'code_drug_J01CA'}
0.56	2	{'eta_53above', 'code_diag_298'}
0.56	2	{'code_drug_N02AX', 'code_drug_J01XX'}
0.56	2	{'code_drug_C09CA', 'code_drug_N03AX'}
0.55	2	{'code_drug_C09CA', 'code_drug_J01XX'}
0.55	2	{'code_drug_R06AX', 'code_drug_C09CA'}
0.54	2	{'code_drug_C09CA', 'code_drug_N02AX'}
0.54	2	{'code_drug_C03CA', 'code_drug_C09CA'}
0.53	2	{'code_drug_C03CA', 'code_diag_298'}
0.53	2	{'code_diag_411', 'code_drug_N03AX'}
0.53	2	{'code_drug_N03AX', 'code_drug_J01CA'}
0.52	2	{'code_drug_R06AX', 'code_diag_298'}
0.52	2	{'code_drug_A11CC', 'code_diag_298'}
0.52	2	{'code_drug_A11CC', 'code_drug_N02AX'}
0.52	2	{'code_diag_411', 'code_drug_R03BA'}
0.52	2	{'code_drug_J01CA', 'code_drug_J01XX'}
0.52	2	{'code_diag_298', 'code_drug_J01XX'}
0.52	2	{'eta_53above', 'code_drug_J01EE'}
0.51	2	{'code_diag_411', 'eta_53above'}
0.51	2	{'code_drug_R06AX', 'code_drug_J01CA'}
0.51	2	{'code_drug_C03CA', 'code_drug_J01CA'}
0.5	2	{'code_drug_A11CC', 'code_drug_J01CA'}
0.5	2	{'eta_53above', 'code_drug_C08CA'}
0.5	2	{'code_diag_411', 'code_drug_R06AX'}
0.5	2	{'code_drug_R03BA', 'code_drug_J01EE'}
0.5	2	{'code_diag_298', 'code_drug_N02AX'}
0.5	2	{'code_drug_C09CA', 'code_drug_A11CC'}
0.63	3	{'eta_53above', 'code_drug_R03BA', 'code_drug_N03AX'}
0.59	3	{'eta_53above', 'code_drug_R03BA', 'code_drug_J01XX'}
0.59	3	{'code_drug_R06AX', 'eta_53above', 'code_drug_R03BA'}
0.58	3	{'code_drug_R06AX', 'eta_53above', 'code_drug_N03AX'}
0.58	3	{'code_drug_N03AX', 'code_drug_R03BA', 'code_drug_J01XX'}
0.58	3	{'code_drug_R06AX', 'code_drug_N03AX', 'code_drug_R03BA'}
0.57	3	{'eta_53above', 'code_drug_R03BA', 'code_drug_N02AX'}

0.56	3	{'code_drug_R06AX', 'code_drug_R03BA', 'code_drug_J01XX'}
0.56	3	{'eta_53above', 'code_drug_N03AX', 'code_drug_J01XX'}
0.55	3	{'code_drug_C03CA', 'code_drug_N03AX', 'code_drug_R03BA'}
0.55	3	{'code_drug_C03CA', 'eta_53above', 'code_drug_R03BA'}
0.55	3	{'code_drug_R06AX', 'eta_53above', 'code_drug_J01XX'}
0.55	3	{'code_drug_R06AX', 'eta_53above', 'code_drug_N02AX'}
0.55	3	{'eta_53above', 'code_drug_N03AX', 'code_drug_N02AX'}
0.54	3	{'code_drug_R06AX', 'code_drug_R03BA', 'code_drug_N02AX'}
0.54	3	{'eta_53above', 'code_drug_R03BA', 'code_drug_A11CC'}
0.54	3	{'eta_53above', 'code_drug_N03AX', 'code_drug_A11CC'}
0.54	3	{'code_drug_N03AX', 'code_drug_R03BA', 'code_drug_N02AX'}
0.54	3	{'code_drug_R06AX', 'code_drug_N03AX', 'code_drug_J01XX'}
0.54	3	{'code_drug_C03CA', 'eta_53above', 'code_drug_N03AX'}
0.52	3	{'code_drug_C03CA', 'code_drug_R03BA', 'code_drug_J01XX'}
0.52	3	{'code_drug_N03AX', 'code_drug_R03BA', 'code_drug_A11CC'}
0.52	3	{'code_drug_R03BA', 'code_drug_N02AX', 'code_drug_J01XX'}
0.52	3	{'code_drug_A11CC', 'code_drug_R03BA', 'code_drug_J01XX'}
0.52	3	{'code_drug_R06AX', 'eta_53above', 'code_drug_C03CA'}
0.52	3	{'code_drug_R06AX', 'code_drug_N03AX', 'code_drug_C03CA'}
0.52	3	{'code_drug_R06AX', 'code_drug_R03BA', 'code_drug_C03CA'}
0.52	3	{'eta_53above', 'code_drug_R03BA', 'code_drug_C09CA'}
0.51	3	{'code_drug_R06AX', 'code_drug_N03AX', 'code_drug_N02AX'}
0.5	3	{'code_drug_C09CA', 'code_drug_N03AX', 'eta_53above'}
0.5	3	{'code_drug_C09CA', 'code_drug_R03BA', 'code_drug_J01XX'}

Cohort 2

Support (s_{min}=0.5)	Length	Frequent Item sets
0.86	1	{'code_drug_A10BA'}
0.79	1	{'code_drug_N02BE'}
0.76	1	{'code_drug_C03CA'}
0.76	1	{'code_drug_J05AB'}
0.74	1	{'code_drug_M04AA'}
0.71	1	{'code_drug_C09CA'}
0.65	1	{'code_drug_C02CA'}
0.65	1	{'code_drug_C08CA'}
0.64	1	{'code_diag_V54'}
0.64	1	{'code_diag_V64'}
0.64	1	{'code_drug_J02AC'}
0.63	1	{'code_drug_N06AB'}
0.63	1	{'code_diag_188'}
0.62	1	{'code_drug_S01EE'}
0.61	1	{'code_drug_N03AG'}
0.6	1	{'code_diag_454'}
0.6	1	{'code_drug_N03AE'}
0.6	1	{'code_diag_820'}

0.6	1	{'code_drug_M01AB'}
0.6	1	{'code_diag_735'}
0.59	1	{'code_drug_B01AA'}
0.56	1	{'code_diag_211'}
0.56	1	{'code_diag_574'}
0.56	1	{'code_drug_C09BX'}
0.56	1	{'code_drug_A07EC'}
0.56	1	{'code_drug_P01AB'}
0.55	1	{'code_drug_B03AA'}
0.55	1	{'code_drug_M01AC'}
0.55	1	{'code_diag_482'}
0.55	1	{'code_diag_550'}
0.55	1	{'code_diag_V56'}
0.54	1	{'code_drug_G04CB'}
0.54	1	{'code_diag_427'}
0.53	1	{'code_diag_571'}
0.52	1	{'code_diag_813'}
0.52	1	{'code_diag_V53'}
0.52	1	{'code_diag_428'}
0.51	1	{'code_drug_S01ED'}
0.51	1	{'code_drug_N02AX'}
0.51	1	{'code_diag_276'}
0.5	1	{'code_drug_N05AH'}
0.5	1	{'code_diag_996'}
0.5	1	{'code_diag_278'}
0.5	1	{'code_drug_R03AL'}
0.68	2	{'code_drug_A10BA', 'code_drug_N02BE'}
0.64	2	{'code_drug_A10BA', 'code_drug_M04AA'}
0.64	2	{'code_drug_A10BA', 'code_drug_J05AB'}
0.64	2	{'code_drug_C03CA', 'code_drug_A10BA'}
0.62	2	{'code_drug_C09CA', 'code_drug_A10BA'}
0.61	2	{'code_drug_J05AB', 'code_drug_N02BE'}
0.6	2	{'code_drug_C03CA', 'code_drug_N02BE'}
0.59	2	{'code_drug_A10BA', 'code_drug_C02CA'}
0.58	2	{'code_drug_M04AA', 'code_drug_N02BE'}
0.57	2	{'code_drug_C03CA', 'code_drug_J05AB'}
0.57	2	{'code_drug_A10BA', 'code_drug_C08CA'}
0.57	2	{'code_drug_C03CA', 'code_drug_M04AA'}
0.57	2	{'code_drug_C09CA', 'code_drug_N02BE'}
0.57	2	{'code_drug_A10BA', 'code_drug_J02AC'}
0.56	2	{'code_drug_C09CA', 'code_drug_J05AB'}
0.55	2	{'code_drug_A10BA', 'code_drug_N06AB'}
0.55	2	{'code_diag_V54', 'code_drug_A10BA'}
0.55	2	{'code_drug_A10BA', 'code_diag_V64'}
0.55	2	{'code_drug_C09CA', 'code_drug_M04AA'}
0.55	2	{'code_drug_J05AB', 'code_drug_M04AA'}
0.54	2	{'code_drug_A10BA', 'code_drug_N03AE'}

0.54	2	{'code_drug_A10BA', 'code_drug_M01AB'}
0.53	2	{'code_drug_A10BA', 'code_diag_188'}
0.52	2	{'code_drug_N02BE', 'code_drug_J02AC'}
0.52	2	{'code_drug_J05AB', 'code_drug_C08CA'}
0.52	2	{'code_drug_N02BE', 'code_drug_C08CA'}
0.52	2	{'code_diag_V54', 'code_drug_N02BE'}
0.52	2	{'code_drug_N02BE', 'code_diag_V64'}
0.52	2	{'code_drug_N02BE', 'code_diag_188'}
0.52	2	{'code_drug_C03CA', 'code_diag_V64'}
0.52	2	{'code_drug_C09CA', 'code_drug_C03CA'}
0.52	2	{'code_drug_A10BA', 'code_diag_735'}
0.52	2	{'code_drug_A10BA', 'code_diag_454'}
0.52	2	{'code_drug_A10BA', 'code_drug_N03AG'}
0.52	2	{'code_drug_A10BA', 'code_drug_S01EE'}
0.52	2	{'code_drug_A10BA', 'code_drug_B01AA'}
0.51	2	{'code_drug_M04AA', 'code_diag_188'}
0.51	2	{'code_drug_P01AB', 'code_drug_A10BA'}
0.51	2	{'code_drug_A10BA', 'code_diag_820'}
0.5	2	{'code_drug_C03CA', 'code_drug_J02AC'}
0.5	2	{'code_drug_N02BE', 'code_diag_454'}
0.5	2	{'code_drug_N02BE', 'code_diag_820'}
0.5	2	{'code_drug_N06AB', 'code_drug_M04AA'}
0.5	2	{'code_drug_C02CA', 'code_drug_N02BE'}
0.52	3	{'code_drug_A10BA', 'code_drug_N02BE', 'code_drug_J05AB'}
0.51	3	{'code_drug_C03CA', 'code_drug_A10BA', 'code_drug_N02BE'}

Cohort 3

Support (s_{min}=0.5)	Length	Frequent Item sets
0.84	1	{'code_drug_N02AX'}
0.82	1	{'code_drug_M04AA'}
0.76	1	{'code_drug_C03EA'}
0.75	1	{'code_drug_A02BA'}
0.73	1	{'code_drug_B01AB'}
0.7	1	{'code_drug_N03AX'}
0.68	1	{'code_diag_813'}
0.68	1	{'code_diag_295'}
0.68	1	{'code_drug_N02AA'}
0.65	1	{'code_drug_J05AB'}
0.62	1	{'code_drug_C07BB'}
0.62	1	{'code_drug_A12AA'}
0.61	1	{'code_drug_B03BB'}
0.6	1	{'code_drug_R03AC'}
0.58	1	{'code_diag_427'}
0.56	1	{'code_diag_V53'}
0.56	1	{'code_diag_518'}

0.56	1	{'code_drug_A12BA'}
0.56	1	{'code_diag_413'}
0.55	1	{'code_drug_J01DC'}
0.55	1	{'code_diag_574'}
0.55	1	{'code_drug_N06AA'}
0.54	1	{'code_drug_A05AA'}
0.53	1	{'code_drug_G03AA'}
0.52	1	{'code_drug_C01DA'}
0.5	1	{'code_diag_618'}
0.5	1	{'code_diag_553'}
0.5	1	{'code_diag_434'}
0.5	1	{'code_diag_727'}
0.71	2	{'code_drug_M04AA', 'code_drug_N02AX'}
0.64	2	{'code_drug_N02AX', 'code_drug_A02BA'}
0.64	2	{'code_drug_M04AA', 'code_drug_A02BA'}
0.63	2	{'code_drug_N02AX', 'code_drug_B01AB'}
0.62	2	{'code_drug_C03EA', 'code_drug_N02AX'}
0.61	2	{'code_drug_M04AA', 'code_drug_B01AB'}
0.61	2	{'code_drug_C03EA', 'code_drug_M04AA'}
0.6	2	{'code_drug_N02AX', 'code_drug_N03AX'}
0.59	2	{'code_drug_N02AX', 'code_diag_813'}
0.58	2	{'code_drug_M04AA', 'code_drug_N03AX'}
0.58	2	{'code_drug_M04AA', 'code_diag_813'}
0.57	2	{'code_drug_N02AA', 'code_drug_N02AX'}
0.57	2	{'code_drug_N02AX', 'code_diag_295'}
0.57	2	{'code_drug_N02AA', 'code_drug_M04AA'}
0.56	2	{'code_drug_C03EA', 'code_drug_A02BA'}
0.56	2	{'code_drug_M04AA', 'code_diag_295'}
0.55	2	{'code_drug_N02AX', 'code_drug_C07BB'}
0.55	2	{'code_drug_A02BA', 'code_drug_B01AB'}
0.55	2	{'code_drug_A02BA', 'code_drug_N03AX'}
0.55	2	{'code_drug_C03EA', 'code_drug_B01AB'}
0.54	2	{'code_drug_C03EA', 'code_drug_N03AX'}
0.54	2	{'code_drug_M04AA', 'code_drug_J05AB'}
0.54	2	{'code_drug_N02AA', 'code_drug_C03EA'}
0.54	2	{'code_drug_A02BA', 'code_diag_813'}
0.54	2	{'code_drug_J05AB', 'code_drug_N02AX'}
0.52	2	{'code_drug_N02AX', 'code_drug_R03AC'}
0.52	2	{'code_drug_C03EA', 'code_drug_J05AB'}
0.52	2	{'code_diag_813', 'code_drug_N03AX'}
0.52	2	{'code_diag_813', 'code_drug_B01AB'}
0.52	2	{'code_drug_A02BA', 'code_diag_295'}
0.52	2	{'code_drug_M04AA', 'code_drug_A12AA'}
0.52	2	{'code_drug_C03EA', 'code_diag_295'}
0.52	2	{'code_drug_C03EA', 'code_diag_813'}
0.51	2	{'code_drug_N02AX', 'code_drug_A12AA'}
0.5	2	{'code_drug_N02AA', 'code_drug_A02BA'}

0.5	2	{'code_drug_B01AB', 'code_drug_N03AX'}
0.5	2	{'code_drug_B03BB', 'code_drug_N02AX'}
0.5	2	{'code_drug_M04AA', 'code_drug_R03AC'}
0.5	2	{'code_drug_M04AA', 'code_drug_B03BB'}
0.5	2	{'code_drug_M04AA', 'code_drug_C07BB'}
0.56	3	{'code_drug_M04AA', 'code_drug_N02AX', 'code_drug_A02BA'}
0.54	3	{'code_drug_M04AA', 'code_drug_N02AX', 'code_drug_B01AB'}
0.52	3	{'code_drug_M04AA', 'code_drug_N02AX', 'code_drug_N03AX'}
0.52	3	{'code_drug_C03EA', 'code_drug_M04AA', 'code_drug_N02AX'}
0.52	3	{'code_drug_M04AA', 'code_drug_N02AX', 'code_diag_813'}

Cohort 4

**Support (s_{min}=0.5)**	**Length**	**Frequent Item sets**
0.8	1	{'code_drug_G04CA'}
0.73	1	{'code_drug_J01CA'}
0.66	1	{'code_drug_C09AA'}
0.66	1	{'code_drug_C09DA'}
0.64	1	{'code_drug_B01AA'}
0.62	1	{'code_drug_C03CA'}
0.61	1	{'code_diag_995'}
0.59	1	{'code_drug_N04AA'}
0.57	1	{'code_diag_153'}
0.57	1	{'code_drug_J02AC'}
0.56	1	{'code_diag_437'}
0.56	1	{'code_drug_C09BX'}
0.55	1	{'code_diag_298'}
0.55	1	{'code_drug_N05AH'}
0.55	1	{'code_diag_250'}
0.54	1	{'code_drug_A10BA'}
0.52	1	{'code_diag_415'}
0.52	1	{'code_drug_C07AG'}
0.5	1	{'code_diag_424'}
0.5	1	{'code_diag_786'}
0.5	1	{'code_drug_C03EB'}
0.5	1	{'code_diag_780'}
0.57	2	{'code_drug_G04CA', 'code_drug_J01CA'}
0.57	2	{'code_drug_C09AA', 'code_drug_G04CA'}
0.53	2	{'code_drug_G04CA', 'code_drug_C09DA'}
0.52	2	{'code_diag_995', 'code_drug_G04CA'}
0.52	2	{'code_drug_C09AA', 'code_drug_J01CA'}
0.51	2	{'code_drug_B01AA', 'code_drug_G04CA'}
0.51	2	{'code_drug_C09DA', 'code_drug_J01CA'}
0.5	2	{'code_drug_C03CA', 'code_drug_G04CA'}
0.5	2	{'code_drug_B01AA', 'code_drug_J01CA'}
